# DX243 counteracts both acoustic trauma-induced reduction in cortical brain oscillations and cochlear synaptopathy

**DOI:** 10.3389/fphar.2025.1673189

**Published:** 2026-01-14

**Authors:** Stefan Fink, Csaba Harasztosi, Li Xie, Stephane Silvente, Pierre Attali, Nicolas Caron, Wibke Singer, Lukas Rüttiger, Marlies Knipper

**Affiliations:** 1 Department of Otolaryngology, Head and Neck Surgery, Tübingen Hearing Research Centre (THRC), Molecular Physiology of Hearing, University of Tübingen, Tübingen, Germany; 2 Department of Otolaryngology - Head and Neck Surgery, Tongji Hospital, Tongji Medical College, Huazhong University of Science and Technology, Wuhan, China; 3 Dendrogenix S.A., Liège, Belgium

**Keywords:** age-related hearing loss, auditory processing, cochlear synaptopathy, cortical oscillations, dendrogenin

## Abstract

**Background:**

With age, even mild acoustic injuries can accumulate - ultimately leading to a characteristic clinical picture of age-related hearing loss with the risk of cognitive decline. Age-related hearing loss is currently attributed to the loss of a most vulnerable auditory fiber type contributing to hearing in background noise. Here, we investigated the potential of a new drug, a more stable chemical variant of Dendrogenin B (DX243) that is predicted to display a protective function on neurite outgrowth, rather than on cell survival.

**Methodology:**

In the rat animal model, DX243 was tested on hearing thresholds, cortical local field potentials (LFP), auditory steady-state responses (ASSR), auditory brainstem responses (ABR), growth function of notched-noise stimuli, inner hair cell (IHC) ribbon numbers, and multi-click pulse responses (MCP) before and after auditory trauma (AT).

**Results:**

No auditory evoked LFP response changes were seen in the prefrontal cortex, visual cortex, or hippocampus. In contrast, a permanent decline of auditory evoked LFP amplitudes in the auditory cortex (AC) was measured 2 weeks after AT. Daily injection of DX243 for 2 weeks prevented the AT-induced decline in LFP activity, a protective effect that continued for 6 weeks following the final injection. Through a protective effect of DX243 on a trauma-induced decline of LFP in response to amplitude-modulated tones >80 Hz, a subcortical origin of the drug-effect was suggested. DX243 showed no effect on hearing sensitivity and did not affect subcortical processing of sound features that depend on phase-locking at the stimulus onset (e.g., near-threshold ASSR responses, close-to-threshold ABR growth functions, or spontaneous cortical LFP responses). However, with the same dose-response dependency as shown for cortical LFP effects, DX243 significantly protected from AT-induced decreases in ABR growth functions for loud sound stimuli and in background noise, protected from AT-induced loss of IHC ribbons and AT-induced decline of fast ABRs with decreasing time intervals.

**Conclusion:**

These findings suggest DX243 as a novel neuroprotective drug, that specifically protects auditory fibers contributing to the coding of the temporal envelope and detection of tones in masking noise, tasks of our auditory senses, both of which, if they fail, lead to age-related speech-in-noise comprehension deficits.

## Introduction

Age-related hearing loss (presbycusis) is a growing problem in aging societies (Peelle and Wingfield, 2016), particularly as it is associated with cognitive decline (Golub et al., 2020; Gregory et al., 2020; Livingston et al., 2020; Livingston et al., 2024; Livingston et al., 2017; Fortunato et al., 2016). It has devastating effects on the everyday life of people who are handicapped in speech recognition, thus risking social isolation (Keithley, 2020). Elderly people often show degraded hearing performance and have difficulties in understanding speech, particularly in noisy environments, features that are linked to changes in cortical responses, as shown in rodents (Gourevitch and Edeline, 2011) and humans (Price et al., 2017; Presacco et al., 2019; Ross et al., 2020; Casarella et al., 2024; Frisina, 2018; Frisina, 2009). Much evidence indicates that, in the noise-exposed ear, distinct peripheral synaptic connections on inner hair cells (IHCs) are the most vulnerable elements (Kujawa and Liberman, 2019). Auditory fibers with low-spontaneous firing rate and high activation threshold (low-SR ANF) are suggested to be more vulnerable for age and noise damage (Monaghan et al., 2020; Bramhall et al., 2019) compared to hair cells (Kujawa and Liberman, 2019) and spiral ganglion neurons (Khan et al., 2005; Nadol and Eddington, 2006).

Newest findings support the notion that the loss of peripheral synaptic connections on IHCs (Kujawa et al., 2011; Liberman and Kujawa, 2017), speech-in-noise discrimination (Guest et al., 2018; Carcagno and Plack, 2022; Apoux and Bacon, 2004; Le Prell et al., 2013; Levy et al., 2015; Vitela et al., 2015; Moore et al., 2017; Motlagh Zadeh et al., 2019; Hunter et al., 2020; Song et al., 2022; Pichora-Fuller et al., 2007; Frisina, 2009; Bajin et al., 2022; Füllgrabe et al., 2014), and cognitive decline (Golub et al., 2020; Hoppe et al., 2022) occur even in subjects with nearly normal audiograms, as based on pure-tone threshold (PTT). This accords with the high prevalence of difficulties in understanding speech in noise among aging populations, that is linked with peripheral and cortical dysfunctions reported to occur in individuals both with and without hearing loss (Frisina and Frisina, 1997; Frisina, 2009; Wong et al., 2009; Price et al., 2019; Liberman and Kujawa, 2017; Kobel et al., 2017; Marrufo-Perez and Lopez-Poveda, 2022; Henry, 2022; Keithley, 2020; Fetoni et al., 2015; Fetoni et al., 2022).

Numerous studies have aimed to find effective and targeted therapeutic treatments to prevent presbycusis (Zhang et al., 2025; Hattori et al., 2025; Watson et al., 2017), or the associated central (Williamson et al., 2020), or peripheral (Rzepakowska et al., 2024) cochlear deficits (Paciello et al., 2020). However, up to now a drug therapy that targets the most vulnerable peripheral and central processing deficits that can occur independently of hearing loss, has evaded discovery. Here, we analyzed the effect of DX243, a 5α-Hydroxy-6β-[3-(4-aminobutylamino)-propylamino]cholestane-3β-ol, that was suggested to induce neurite outgrowth on various cell lines as PC12 and P19 cells *in vitro* (de Medina et al., 2009). In a guinea-pig model, compared to no DX243 treatment, cochlear implant efficacy in the presence of DX243, as measured as the electrical responsiveness of the auditory nervous system, was related to the functional, but not the anatomical preservation of the spiral ganglion neurons. This suggested that it is unlikely that DX243 affects neuronal cell survival, but rather cell outgrowth (Fransson et al., 2015).

As age-related primary neural degeneration was previously linked to accumulating acoustic injury over the life-time (Wu et al., 2021; Fetoni et al., 2022), we considered testing the effect of DX243 in a first approach on acoustic injury. The influence of DX243 was analyzed *in vivo* following acoustic injury regarding (i) central and (ii) peripheral processing deficits. (i) For the analysis of central processing, the evaluation of oscillatory components of neural cortical responses promised a deeper understanding of speech-in-noise problems (Price et al., 2019). Indeed, altered neural oscillations or permanent receptive field changes have been shown following aging (Allison et al., 1984; Bertoli et al., 2002; Peelle and Wingfield, 2016; Zhao et al., 2024; Price et al., 2019; Wostmann et al., 2015; Ross et al., 2020), but also following acoustical, mechanical, or drug-induced trauma (Calford et al., 1993; Norena et al., 2003; Robertson and Irvine, 1989; Yang et al., 2007; Fetoni et al., 2015).

Only recently, altered cortical local field potentials (LFP) identified at distinct oscillation frequencies in the auditory cortex (AC) have been linked to specific cochlear synaptopathy in animal models (Marchetta et al., 2024). Using electroencephalographic (EEG) event-related potentials, moreover, a connection between reduced EEG amplitudes and latency shifts and distinct cochlear synaptopathy and deficits in stimulus onset coding in humans have been demonstrated (Dapper et al., 2025). Finally, it was explicitly shown that the activation of evoked brain oscillations in the gamma band (≥25 Hz) is associated with enhanced stimulus-induced performance (Chen et al., 2017; Kim et al., 2016). This leads to enhanced response reliability, decreased signal variability, and improved reliability of information processing through an improved signal-to-noise ratio (Cardin et al., 2009; Sohal et al., 2009; Zhu et al., 2015). Together, these observations motivated us to choose LFP measurements in the rat cortex to assure a higher translational impact of potentially identified DX243 effects. Cortical LFP and their spectral activity were analyzed in the present study, as described in a previous study in mice (Marchetta et al., 2024). To obtain an insight into the contributions of phase-locked synchronous responses, we furthermore analyzed stimulus-evoked (phase-coherent), induced (phase-incoherent) LFP, and spontaneous (resting-state) brain oscillations, as well as amplitude-modulated auditory steady-state responses. (ii) Having noted that de-afferentation of surviving IHCs following acoustic trauma or aging may be a major contributor to auditory dysfunction, we also analyzed IHC synaptic damage in more detail. Accordingly, presynaptic ribbon-like structures stained by antibodies directed against the RIBEYE protein CtBP2 at the base of IHCs were analyzed as described by Ruttiger et al. (2017). Contributions of noise-sensitive and of resistant auditory fiber subtypes were analyzed through ABR growth functions of evoked auditory responses in the presence and absence of notched-noise. ABR responses to multi-click pulses of decreasing time intervals were also analyzed in the absence and presence of AT and DX243. All experiments were performed in the Wistar rat animal model as an accepted presbycusis animal model (Lippincott and Rarey, 1997; Rüttiger et al., 2007).

We observed that DX243 significantly prevented an AT-induced decline of local cortical field potentials, even in response to amplitude-modulated tones of >80 Hz modulation frequencies, suggesting a presumptive subcortical origin of the effect. DX243 did not affect hearing thresholds *per se*, nor spontaneous brain oscillations or near-threshold ASSR or ABR growth functions. These have in common a dependence on phase-locked synchronized responses at stimulus onset, and thereby possibly on high-spontaneous firing auditory fibers that have sensitive activation thresholds (high-SR ANF). In contrast, the results showed a DX243-induced preferential preservation of the ABR growth function for high sound levels, explicitly including those functions that were robust to background noise. Parallel to this, a DX243-induced preservation of IHC ribbon number despite AT, and a DX243-induced sustaining effect on multi-click ABR responses with decreasing time intervals were observed despite AT, supporting the idea that DX243 counteracts cochlear synaptopathy. This finding is discussed in the context of a potential therapeutic window for treatment with DX243, which could counteract age-related peripheral and central damage caused by the accumulation of acoustic injuries. Specifically, the age-related consequences of the loss of the most vulnerable low-spontaneous rate auditory fibers are considered to be a target of DX243.

## Materials and methods

### Animal care

Female Wistar rats were purchased from Charles River Laboratories (Sulzfeld, Germany) and were housed for up to 6 months in the animal-care facility of the institute, where 50–60 dB SPL noise levels were not exceeded. The care and use of animals was approved by the University of Tübingen, Veterinary Care Unit, and the Animal Care and Ethics Committee of the regional board of the Federal State Government of Baden-Württemberg, Germany, and followed the guidelines of the EU Directive 2010/63/EU for animal experiments.

### Housing conditions

Animals were housed in small social groups (4-5 rats), that were stable throughout the experiment. Cages were equipped with nesting material and a hiding place. Conditions in the animal room are set according to the standard operating procedure of the Test Facility, and were as follows: Temperature: 25 °C; Light/Dark cycle: 12 h/12 h, light phase starts at 6 o´clock am; Food and water were available *ad libitum* throughout the study.

### Preparation of the test item and application

DX243 was provided by the company Dendrogenix (Liège, Belgium). 20 mg of DX243 were dissolved in 100 mL of 0.9% NaCl (Fresenius Kabi, Bad Homburg, Germany) and if necessary diluted to the desired concentration using 0.9% NaCl. Solutions were prepared before the first application and were stored in the refridgerator during the individual ongoing experiments.

### Anesthesia

For the functional hearing tests and for the animals subject to traumatic sound-exposure, anesthetia used fentanyl dihydrogencitrate (0.005 mg/kg; Fentanyl, Ratiopharm, Ulm, Germany), medetomidine hydrochloride (0.15 mg/kg: Sedator, Eurovet Animal Health B.V., Aulendorf, Germany), and midazolam (2.0 mg/kg: Midazolam-hameln, Hameln Pharma Plus GmbH, Hameln, Germany). After the tests and the exposure, anesthesia was antagonized using naloxon hydrochloride (0.12 mg/kg: Naloxon-hameln, Hameln Pharma Plus GmbH, Hameln, Germany), flumazenil (0.2 mg/kg: Flumazenil-Kabi, Fresenius Kabi, Bad Homburg, Germany) and atipamezol hydrochloride (0.75 mg/kg: Antisedan, Orion Pharma, Espoo, Finland, distrib. by Zoetis Inc., Parsippany, NJ, United States).

### Study design

As for all (ABR) hearing measurements, drug-application procedures and the acoustic trauma protocol were previously established in female Wistar rats (Rüttiger et al., 2013; Möhrle et al., 2016; Bing et al., 2015; Singer et al., 2013; Singer et al., 2018; Jaumann et al., 2012), we here evaluated the potential drug effects of DX243 in female rats. In total, 53 female Wistar rats, 2–3 months old at arrival, were used in this study. Animals acclimatized to the animal facility and handling by the experimenters for 2 weeks. One week after the first baseline hearing measurements, animals were either exposed to a traumatizing sound stimulus (Acoustic Trauma (AT) treatment, see method section) or were sham treated (Sham), undergoing the same treatment as AT animals just without sound stimulation. On the same day as the exposure, the daily subcutaneous administration of either vehicle (0.9% NaCl solution) or DX243 was started and repeated daily for another 13 days. DX243 was administered in different doses to three groups: 0.01 mg/kg, 0.05 mg/kg or 0.1 mg/kg body weight. In the first cohort, the 14-day long treatment was followed by the final hearing measurements and follow-up EEG recordings (Supplementary Figure S1A). For 16 out of 35 animals, the application was extended until the day before of the EEG measurement. In the second cohort, the 14^th^ injection was followed by a 6-week long (56 days) wash-out period before the final hearing and EEG measurements were performed (Supplementary Figure S1B). For both cohorts (Cohort 1: 14 days s.c., and Cohort 2: 14d + 6 weeks), animals were randomly assigned to one of five groups named Sham+Veh (N = 9 + 4), AT+Veh (N = 9 + 5), AT+0.01 mg/kg (N = 6 + 0), AT+0.5 mg/kg (N = 6 + 5), AT+0.1 mg/kg (N = 5 + 4) (Supplementary Table S1).

### Auditory brainstem responses (ABR)

Hearing thresholds were determined by measurement of ABR as previously described (Ruttiger et al., 2017). In brief, subdermal silver-wire electrodes were inserted at the vertex (reference, negative) and ventrolateral to the measured ear (active, positive) and the back (ground) of the anesthetized rats. ABR to click, broadband noise-burst, and pure-tone stimuli between 2.0 and 32 kHz (in 2 steps per octave) were measured. Stimulus sound pressure levels were typically 20–100 dB SPL, presented in steps of 5 dB. The hearing threshold was determined by the lowest sound pressure that produced potentials visually distinct from noise. Stimuli were generated by a multi-function IO-Card (PCI-6251, National Instruments, Austin, Texas, USA), housed in an IBM compatible computer.

The click stimulus used in the present experiment was a broadband stimulus (Supplementary Figure S2A). The plateau ranged from 1 to 6 kHz, with a 30 dB roll-off from 6 to 45 kHz. Local minima were observed at 10, 20, 30, and 40 kHz of the spectrum. Apart from the click stimulus, a broadband noise-burst stimulus (noise stimulus) was used. The duration of the noise stimulus was 1 ms with no onset and offset ramps. The spectral plateau ranged from 1 to 35 kHz with a 30 dB roll-off from 35 to 65 kHz (Supplementary Figure S2B). Compared with the click stimulus, the noise-burst stimulus contained more energy at higher frequencies (>10 kHz), which was confirmed by a higher center frequency (7.9 kHz) of the spectrum. From the ABR waveforms evoked by click- and noise-burst stimuli, the amplitudes and latencies of ABR wave I and ABR wave IV were extracted. The time interval was defined for wave I as: 0.9–1.4 ms, and for the wave IV: 3.5–5.8 ms. A customized computer program was used for the extraction of ABR peaks. The wave growth functions were calculated for increasing stimulus levels with reference to the corresponding wave thresholds (Ruttiger et al., 2017).

### Acoustic trauma (AT)

Anesthetized animals were exposed for 60 min either to a continuous 10 kHz pure-tone stimulation presented at 116 dB SPL, or to a sham treatment without sound stimulation. As previously described (Ruttiger et al., 2017), the traumatizing sound was delivered in a reverberating chamber (a chamber of approx. 50 × 50 × 50 cm with tilted, non-parallel walls to achieve a mostly homogeneous sound field) and equipped with seven loudspeakers (1x Visaton DR45N, Visaton, Haan, Germany, and 6x Monacor MPT-005, Conrad Electronic, Hirschau, Germany). All acoustic stimuli were calibrated at the head level of the animal. During the 60 min exposure or sham treatment, animals were continuously moved circular on a turntable within the chamber to achieve homogeneous exposure of both ears. Health and anesthesia status were controlled every 30 min. Animals in the sham-exposed group were treated identically with the loudspeakers turned off.

### Multi-click pulse (MCP) measurement

To address impairment in fast synaptic transmission, a series of repeated click stimuli (trains of two, three, four, and five clicks) with decreasing interstimulus time intervals (ISI, range 32 ms – 1 ms) was presented. The stimulus intensity was 30 dB above hearing threshold (sensation level, SL). The decreasing ISI and the increasing number of clicks per stimulus train challenged the synaptic transmission at the IHCs synapses, which resulted in greater response time variances of post-synaptically generated auditory nerve action potentials, and therefore smaller ABR response amplitudes. Click-response amplitudes were normalized to the first click-response at each ISI of the train and separated from the previous pulse response by subtracting the response so the click train containing n-1 click stimuli (e.g., the response to the click number 5 in the 5-click stimulus train, Click5 = Click5 minus Click4; and correspondingly for the other click train series). To analyze MCP signals, the normalized data were fitted using a double-Boltzmann function (Equation 1):

$$f=\left(p/\left(1+\exp\left(-\left(t-m1\right)/s1\right)\right)+\left(1-p\right)/\left(1+\exp\left(-\left(t-m2\right)/s2\right)\right)\right)$$ (1)

where t is the time, p is the initial part (first portion of the two components of the double-Boltzmann function) while 1-p is the second portion m1, m2 and s1, s2 are the mid-point and the slope of the components, respectively. The coefficients were fitted in Microsoft Excel using the Solver Add-in.

### Auditory responses in notched-noise masking

**FIGURE 1:**
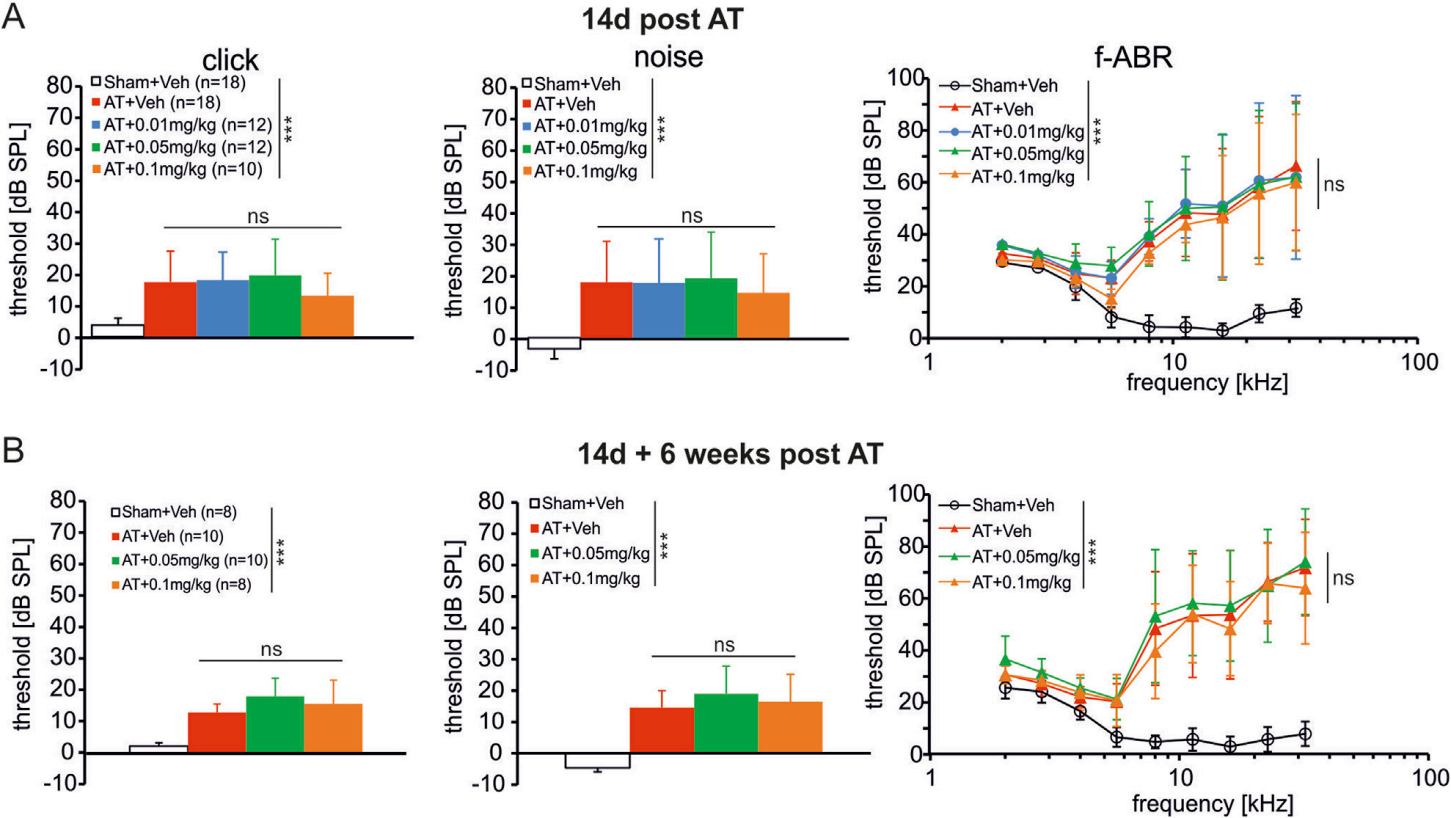
Hearing function 14d post AT **(A)** and 14d post AT + 6 weeks **(B)**. All sound exposed rats show an increase in hearing threshold 14d post AT **(A)** and 14d post AT + 6 weeks **(B)**, especially for frequencies > 5 kHz (f-ABR). A treatment specific effect was not observed in these measurements. Click and noise ABR: Kruskal–Wallis test, Dunn’s multiple comparison test; f-ABR: Two-way ANOVA, Dunnett´s multiple comparison test.

To isolate responses of auditory nerve fibers that are sensitive within a particular auditory frequency and stimulus level range of hearing, we recorded ABRs to 8 kHz pure-tones presented in a spectrally notched noise masker (notched-noise). As seen in the f-ABR responses of the AT-treated groups (Figure 1, right panels), the permanent threshold shift caused by the 10 kHz pure tone acoustic trauma started at around 6 kHz and rose steeply toward higher frequencies. Therefore, we chose the lower 8 kHz frequency region to assure that ABR responses were still measurable.

The notched-noise stimulus had a broadband noise spectrum with a 0.5 octave spectral gap centered at 8 kHz. ABRs were recorded using two sets of measurements: (i) the 8 kHz pure tone bursts presented alone, and (ii) the notched-noise and the 8 kHz pure tone bursts presented concurrently at the same stimulus level. Stimulus sound-pressure levels were typically 0–99 dB SPL, presented in 3 dB steps. For each condition, peak-to-peak amplitudes were extracted within a 4 ms time window, including both the ABR wave I and wave IV responses. The difference between the 8 kHz response amplitude for both measurements (8 kHz with and without notched-noise stimuli) was reported as a measure for the number of fibers being masked by (i.e., were sensitive to) the notched-noise masker.

### Electroencephalographic recordings (EEG)

#### Local field potentials

In response to auditory stimuli, cortical local field potentials (LFP) were recorded in the auditory cortex (AC), prefrontal cortex (PFC), visual cortex (V1), and hippocampus (HC) with a reference electrode placed on the cerebellum (Cb). Amplitude-modulated auditory steady-state responses (ASSR), as well as stimulus-evoked (phase-coherent), induced (phase-incoherent), and spontaneous (resting-state) brain oscillations were analyzed (Marchetta et al., 2024).

For EEG recordings, either pure tone stimuli (at 4.66, 5.66, 6.66, 10.3, 11.3, 12.3, 21.7, 22.7, and 23.7 kHz) or ASSR stimuli were used to elicit local field potential (LFP) responses. In addition to increasing levels from 10 to 100 dB SPL, a fixed 70 dB SPL level was used for modulation frequencies ranging from 5 to 2048 Hz (ASSR stimulus pattern, see Supplementary Figure S2C).

#### Evoked- and induced responses

Cortical LFP analyses comprised resampling of the digital recordings at 3000/s (cutoff frequency 2400 Hz, cutoff width 600 Hz, using the EEGLAB (V2022.0, Swartz Center for Computational Neuroscience, CA, USA) resample function) and artifact detection and rejection of individual repetitions (trials or epochs) by a variance-amplitude criterion, rejecting the 5% trials with the highest variance. Segmentation and transformation of the first 100 of 120 ms stimulus duration (inter-stimulus interval 387 ms) into the frequency space was performed by Fast Fourier Transformation (FFT, rectangular window). The LFP measured as peak-to-peak (the minimum and maximum potential) amplitudes within predefined time intervals were computed for evoked and induced oscillations resulting from phase-coherent/phase-incoherent averaging. Evoked responses (phase-locked to the stimulus) were computed by averaging EEG epochs in the time domain across multiple trials before the FFT. For the induced responses (not phase-locked), a time-frequency decomposition was applied to each trial that was thereafter averaged across trials. No adjustments by subtraction of the evoked and baseline response parts were applied, thus we report here for the induced responses, the overall spectro-temporal activity in the records. The frequency domain was evaluated for the frequency bands alpha (5–15 Hz), beta (15–25 Hz), low-gamma (25–35 Hz), mid-gamma (35–65 Hz), and high-gamma (65–125 Hz).

#### Resting state activity

Approximately 17 min of resting state recording were subdivided into approx. 1,000 epochs of 1 s. The Fourier transform of individual epochs was calculated with Matlab (Version R2021b). The resulting absolute values of the FFT of individual epochs were averaged.

#### Surgery and electrode placement

The surgical method was adopted from the method established for mice (Marchetta et al., 2024). In brief, rats were anesthetized and laid on a pre-warmed (37 °C) resting pad. Local anesthesia was applied by injecting 20–40 μL Xylocain 2% (AstraZeneca, Wedel, Germany) at the sites of surgical incisions. Breathing was continuously supported by oxygen flow to the nostrils. The skin and the connective tissue were removed above the parietal, occipital, and temporal part of the skull so that a patch of approx. 1.5 × 1.5 cm of the bone was exposed. For the reference electrode, a 0.6 mm hole was drilled in the skull 11 mm posterior to bregma (Supplementary Figures S3A, R) above the cerebellum. The electrode was a custom-made silver wire electrode (diameter = 0.140 mm) insulated by varnish and silicone and ending in a small silver bead (ball electrode). Two additional ball electrodes were placed on the cortex’s surface after drilling holes above the left prefrontal cortex (0.5 mm lateral, and 3.7 mm anterior to bregma, Supplementary Figures S3A (1+), S3E) and the left visual cortex (4.5 mm lateral, and 7.1 mm posterior to bregma, Supplementary Figures S3A (2+), S3B). Shaft electrodes were inserted at the given locations for local field potentials of the right AC (6.5 mm lateral, 4.8 mm posterior, and 3.5 mm ventral to bregma, Supplementary Figures S3A (3+), S3D), and the left hippocampus (3 mm lateral, 4.16 mm posterior, and 2.7 mm ventral to bregma, Supplementary Figures S3A (4+), S3C). Shaft electrodes were custom made by inserting and fixing a 0.140 mm silver wire into a 27G cannula used as guiding shaft. Electrodes were fixed to the bone with Histoacryl (B. Braun Surgical S.A., Rubi, Spain). Coordinates for the electrodes were chosen according to the Rat Brain Atlas (Paxinos and Watson, 1986, second Edition) and were adapted to the size (estimated from bodyweight) of the animal (Yang et al., 2018). Electrodes were connected to a head stage (LabRat AC16LR, Tucker Davis Technologies, Alachua, FL, USA) connected to a programmable gain amplifier (PGA16 Rev.B, Multichannel Systems MCS, Reutlingen, Germany). The PGA16 supports frequency bands between 5 Hz and 5 kHz. It was used at a gain of 5,000 and connected to a multi-I/O measurement card (NI PCIe-6321, National Instruments, Austin, TX, USA) housed in a personal computer.

### Immunohistochemistry and ribbon-synapse counting

Immunohistochemistry was performed on cryosectioned cochleae as previously described in detail (Singer et al., 2016). Antibodies were used against C-terminal binding protein 2 (CtPB2/RIBEYE, ARP, Waltham, MA, United States, diluted 1:2000) to detect synaptic ribbons (Khimich et al., 2005), and neurofilament 200 (NF200, Sigma-Aldrich, Taufkirchen, Germany, diluted 1:3000), to detect afferent fibers. Primary antibodies were detected using Cy3 (1:1500, Jackson Immuno Research Laboratories, West Grove, PA, United States) or Alexa488 (1:500, Invitrogen Molecular Probes, Paisley, United Kingdom) secondary antibodies.

Samples were viewed as previously described (Zampini et al., 2010) using an Olympus BX63 microscope equipped with epifluorescence illumination (×100 objective, NA = 1.35) and a motorized z-axis. Images were acquired using a CMOS camera (ORCA-Flash4.0 LT3 Digital, Hamamatsu, Herrsching am Ammersee, Germany) and the imaging software Cell Sense (OSIS GmbH, Münster, Germany). In brief, CtBP2/RIBEYE immunopositive spots were imaged over a distance of 15 µm with the complete coverage of the IHC nucleus along the z-axis (z-stack). Typically, z-stacks consist of 30 layers with a z-increment of 0.49 μm. For each layer one image per fluorochrome was acquired. Z-stacks were 3-dimensionally deconvoluted using Cell Sense’s deconvolution module using the Nearest-Neighbor algorithm (OSIS GmbH, Münster, Germany).

### Statistical analysis

Statistical analyses were carried out with GraphPad Prism Software 10.5.0 (Boston, MA, USA). We used an alpha level of 0.05 for all statistical tests; n.s. (ns) not significant, for significances: *p < 0.05; **p < 0.01, ***p < 0.001. ABR measurements are analyzed by One-way ANOVA (click-, noise-ABR) or Two-way ANOVA (f-ABR), followed by Dunnett´s multiple comparison test (compare the mean of each group with the mean of the Sham+Veh group) or by Kruskal–Wallis test, followed by Dunn´s multiple comparison test. When the comparison of the mean of each group with the mean of every other group was performed, the Tamhane’s (after One-way ANOVA) or Tukey’s multiple comparison test (after Two-way ANOVA) was applied. ABR wave amplitudes, latencies, and ratios were analyzed by Two-way ANOVA, followed by Dunnett´s multiple comparison test (mean of each group vs. mean of the Sham+Veh group), or Tukey’s multiple comparison test (comparison of the mean of each group with the mean of every other group). IHC ribbon numbers were analyzed by One-way ANOVA, followed by Tukey’s multiple comparison test. Amplitude growth of the ABR response within the selected post-stimulus onset time window in response to unmasked 8 kHz tones, and the response with the simultaneously presented notched-noise masker was calculated from individual ears and compared by Two-way ANOVA and *post hoc* Sidak’s multiple comparison test for effects of treatment and stimulus level. After significant ANOVA test results, statistical differences were confirmed by *post hoc* testing. Significances are indicated in figures by asterisks. Here, if not otherwise stated, the results of the Dunnett’s *post hoc* test (multiple comparison test against one control [Sham+Veh group]) are shown. If no asterisk is highlighted, no statistical difference between groups (ns) were found. If not otherwise stated, figures are presented as group means ± SD. “N” denotes the number of animals, while “n” represents the number of evaluated ears.

## Results

### DX243 does not prevent auditory trauma-induced hearing thresholds shifts

DX243 is hypothesized to act through a neurotrophic pathway, but the specific target is still not known. It is, however, currently assumed that DX243 exhibits no effect on the survival of sensory or neuronal cells (De Medina et al., 2009; Fransson et al., 2010; Fransson et al., 2015; Maruyama et al., 2008). To validate this hypothesis, we determined the hearing thresholds of rats before, and 14 days after, acoustic trauma and treatment with DX243.

There was a significant and permanent hearing threshold shift (PTS) 2 weeks (Figure 1A) and 8 weeks (Figure 1B) after AT induced by click or noise stimuli (*Kruskal–Wallis test, Dunn´s multiple comparison test,* p = 0.001, n = 10–18 ears), regardless of the DX243 dosage used (n = 8–12 ears). The PTS for the broadband click and noise stimuli was confirmed when DX243 was tested on AT-induced elevation of hearing thresholds for narrow-band (or: pure-tone) tone bursts (f-ABR) 2 weeks after the start of injections (Figure 1A, f-ABR), or after 6 weeks wash-out following the last injection with either vehicle or DX243 (Figure 1B, f-ABR). As expected, no effect of a daily injection of any DX243 dose on PTS was observed, neither for click stimuli (Figures 1A,B, click), nor noise stimuli (Figures 1A,B, noise), nor when tested using narrow-band (or: pure-tone) tone bursts (Figures 1A,B, f-ABR).

Because outer hair cells (OHCs) are suggested to define the thresholds of auditory brainstem responses (Dallos and Harris, 1978), this conclusively indicates that the applied doses of DX243 did not impact AT-induced impairments of mechano-electrical properties of OHCs and hearing thresholds *per se*.

### DX243 counteracts auditory trauma-induced reduction of evoked LFP in the auditory cortex

Local field potentials (LFPs) were recorded from the AC and HC through insertion electrodes, using the electrode positions as previously described (Li et al., 2021), and from the PFC (Tseng and Han, 2021) and V1 (Marchetta et al., 2024) on-scalp, using single-shank linear silicon electrodes (electrode position, see Supplementary Figure S3). The electrode positions of the AC and HC (insertion electrode) and PFC and V1 were confirmed after the experiment by histology.

**FIGURE 2:**
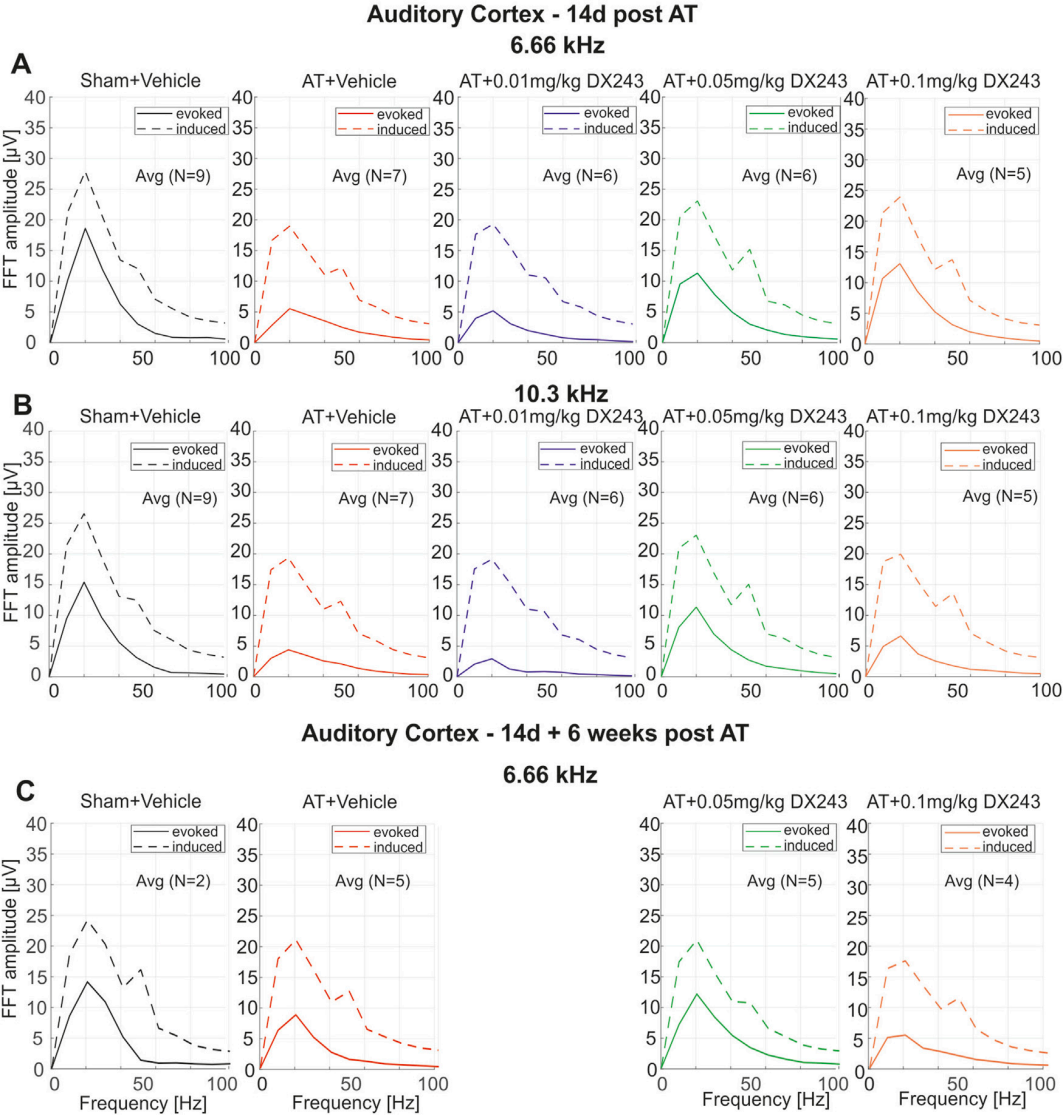
EEG spectral response amplitudes 14d after exposure **(A,B)** and 14d post AT + 6 weeks **(C)**. Evoked (solid lines) and induced (dashed lines) responses of the different treatment groups (left to right) at **(A)** 6.66 kHz and **(B)** 10.3 kHz auditory stimulation 14d after exposure and **(C)** at 6.66 kHz auditory stimulation 14d post AT + 6 weeks after exposure. For all stimulus frequencies, the sham exposed group (**A-C**, left, black solid/dashed lines) shows the highest response, while the AT+Veh [**(A–C)**, red solid/dashed lines] and the AT+0.01 mg/kg DX243 [**(A,B)** middle, blue solid/dashed lines] groups show the two lowest response amplitudes. The AT+0.05 mg/kg DX243 group [**(A-C)**, green solid/dashed line] shows the highest response amplitudes of the three dosages used and of all sound exposed groups, indicating a partly protective effect of 0.05 mg/kg DX243 on EEG responses.

The LFP responses were studied as a function of pure-tone frequencies between 4 and 23 kHz at 90 dB SPL 2 weeks after AT, or after sham exposure and a daily injection of 0.01, 0.05, or 0.1 mg/kg DX243 or vehicle at the AC (Figures 2A,B), the V1 (Supplementary Figure S4A), HC (Supplementary Figure S4B) and PFC location (Supplementary Figure S4C), or alternatively, 6 weeks after the last DX243 injection (Figure 2C).

To uncover possible connections between peripheral deficits (evoked responses) and altered cortical brain oscillations (induced responses), evoked brain oscillations (frequency specific responses phase-locked to the stimulus presentation) were distinguished from induced brain oscillations (frequency specific responses not phase-locked) (David et al., 2006; Galambos, 1992; Tallon-Baudry et al., 1996; Singer and Gray, 1995).

When evoked LFP amplitudes were compared between the groups (Figure 2), it became evident that in sham-vehicle-treated animals, a strong evoked event-related potential in the AC in response to 6.6 and 10.3 kHz auditory stimuli was seen at 2 weeks (Figures 2A,B, solid line), and as shown for 6.6 kHz 6 weeks after the last injection (Figure 2C, solid line). The LFP response was much weaker in the AT-treated animals (Figures 2A–C AT). In the other brain regions (V1, HC and PFC), no significant change in auditory evoked LFP amplitudes were seen in response to 10.3 kHz stimuli (Supplementary Figure S4A–C), or in response to the other frequencies tested (not shown). This confirms the specificity of an auditory stimulus-evoked event in the AC and its decline 2 and 8 weeks after AT. Neither AT nor one of the DX243 treatments exhibited a strong effect on induced responses (Figures 2A–C, dotted line), or altered the evoked or induced responses in V1, HC, or PFC (Supplementary Figure S4). By contrast, in the AC, DX243 treatment prevented the trauma-induced reduction of LFP, with a maximal effect 2 weeks after daily injection with the 0.05 mg/kg DX243 dose (Figures 2A,B). Even 6 weeks after the last DX243 application, a protective effect was still visible, as shown for 6.6 kHz evoked responses (Figure 2C).

**FIGURE 3:**
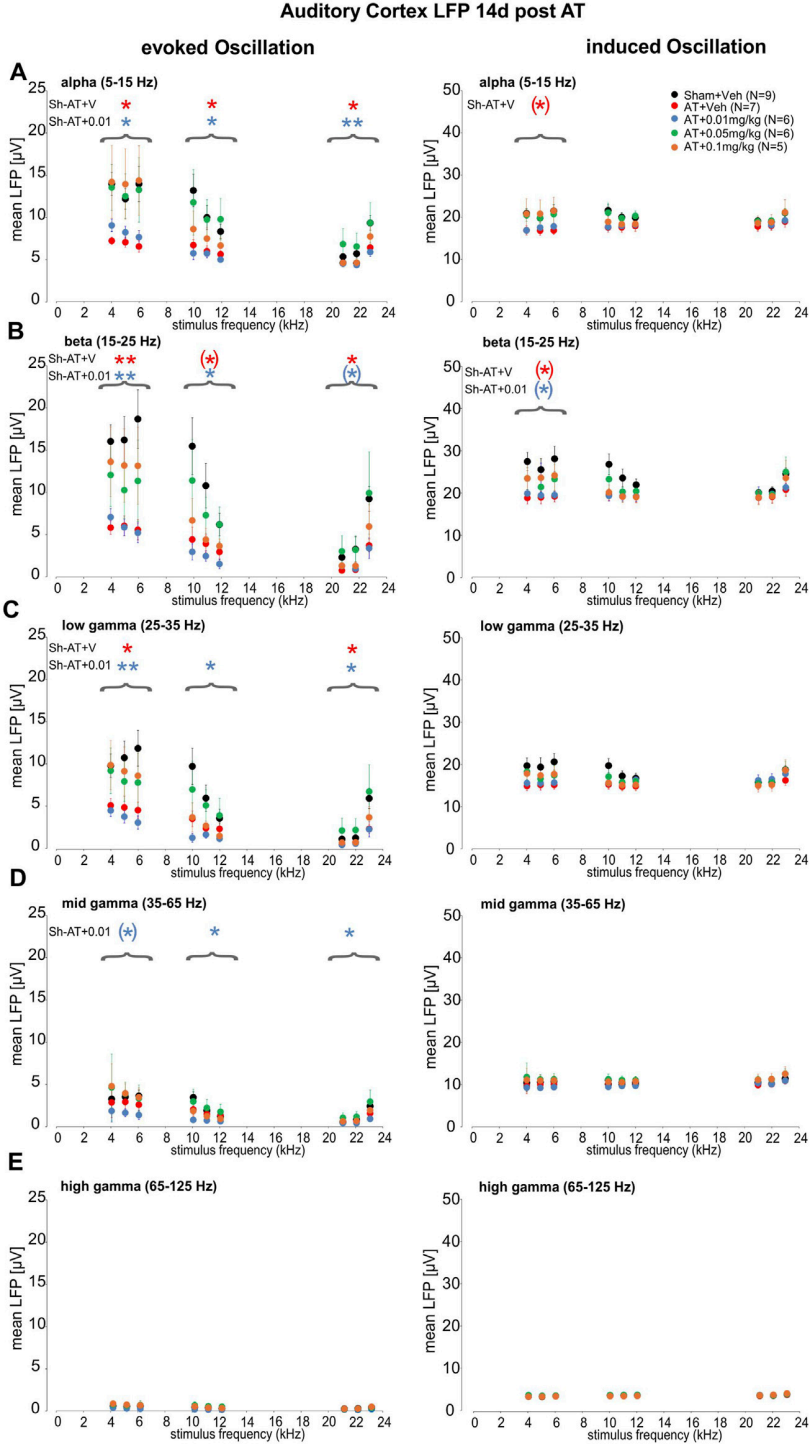
Evoked and induced LFP responses for alpha **(A)**, beta **(B)**, and low-**(C)**, mid- **(D)**, high- **(E)** gamma EEG frequency bands in the auditory cortex. Evoked (left panels) and induced (right panels) LFP activity (mean LFP in μV) in response to low- (4.66–6.66 kHz), middle- (10.3–12.3 kHz), and high-frequency stimuli (21.7–23.7 kHz). The effect of auditory-stimulus frequencies on the different oscillation bands **(A–E)**: **(A)** alpha oscillations (5–15 Hz), **(B)** beta oscillations (15–25 Hz), **(C)** low-gamma oscillations (25–35 Hz), **(D)** mid-gamma oscillations (35–65 Hz), and **(E)** high-gamma oscillations (65–125 Hz) were analyzed by comparing the different treatment groups to the Sham+Veh group. Analyzed by Two-way Repeated Measurement ANOVAs. Evoked (left) and induced (right) LFP responses in AT+Veh (AT+V, red) and AT+0.01 mg/kg DX243 (AT+0.01, blue) were smaller in the alpha, beta, and low-gamma oscillatory bands **(A–C)**. Statistical significance for the three (low-, middle-, and high-) frequency groups (brackets) in Sidak’s multiple comparisons test compared to Sham + Veh (Sh, black) is indicated by red and blue asterisks for AT + V and the AT+0.01, respectively. For the same oscillatory bands **(A–C)**, the AT+0.05 mg/kg DX243 treated group (green) almost reached control levels (Sham + Veh), most consistently at stimulus frequencies ≤ ∼12 kHz. A similar trend was observed for the AT+0.1 mg/kg DX243 treated group (orange) for stimulus frequencies ≤6.66 kHz. Not quite significant (*) 0.05 < p < 0.1.

To further specify and quantify the observed differences in LFP amplitudes following AT and treatment with DX243, we performed time-frequency analysis of the evoked (Figure 3, left) and induced (Figure 3, right) mean LFP amplitude shown for the AC in response to sound stimuli between 4 and 23 kHz and averaged for distinct EEG oscillations (frequency bands). Evoked and induced LFP activity is shown for the alpha band (5–15 Hz) (Figure 3A), beta band (15–25 Hz) (Figure 3B), low-gamma band (25–35 Hz) (Figure 3C), mid-gamma band (35–65 Hz) (Figure 3D), and high-gamma band (65–125 Hz) (Figure 3E) for the different treatment groups (circle color). We observed that 2 weeks after AT and vehicle treatment (Figure 3, red), as well as 2 weeks after AT and treatment with the lowest DX243 dose used of 0.01 mg/kg (Figure 3, blue), the evoked LFP responses were smaller, particularly in the alpha, beta, and low-gamma band (Figures 3A–C). Statistical significance between Sham+Veh and AT was reached mainly below auditory stimuli in the best-frequency hearing range of the rat (<12 kHz) as statistically tested by group-specific *post hoc* analysis (Supplementary Table S2). In contrast to this, and most consistently at stimulus frequencies ≤12 kHz AT+0.05 mg/kg and ≤6 kHz for AT+0.1 mg/kg DX243, oscillation bands nearly reached the level of the Sham+Veh control group (Figures 3A–C, green, left). For the alpha and beta band, the same trend was also observed for induced LFP activity (Figures 3A–C, green, right). Remarkably, a protective effect was seen in extended high-frequency (EHF) regions, nearly reaching statistical significance for responses to 23 kHz auditory stimuli (Figures 3A–C, green, 23 kHz). This protective effect would be worth validating in future studies, considering the observation from a human study that showed that the EHF regions influenced the peripheral and cortical processing of low-frequency auditory information (Schirmer et al., 2024; Dapper et al., 2025, see discussion).

To further determine whether the source of the reduced sound-evoked LFP response in AT and the presumed protection by DX243 originated from a decline of synchronized phase-locked responses in cortical or subcortical auditory nuclei, we analyzed the signal-envelope-following responses to ASSR stimuli. ASSRs reflect the summation of phase-locked (synchronized) activity from multiple generators of neural stimuli within the auditory system, including the cochlea, auditory nerve, inferior colliculus, and AC (Lu et al., 2022). The contributions of ASSR sources in response to lower (10–40 Hz) modulation rates are dominated by cortical components (Lu et al., 2022), while ASSRs in response to higher (approx. >80 Hz) modulation rates are dominated by subcortical components (Lu et al., 2022; Kuwada et al., 2002).

ASSRs were evoked by a pure-tone stimulus with a carrier frequency of 8 kHz and presented at 70 dB SPL. The carrier was sinusoidally amplitude-modulated (100% depth), with modulation frequencies (mf) between 5 and 2048 Hz (Supplementary Figure S5A). After Fast Fourier transformation (FFT), the spectral activity at mf (Hz) was compared between the treatment groups as a function of mf (Supplementary Figure S5A). A profound decline of evoked ASSR amplitudes was observed by AT in all groups for mfs above 16 Hz (Supplementary Figure S5A), without any significant difference between the vehicle and any of the 3 DX243 treatment doses (Supplementary Figure S5A; Supplementary Table S2). However, we observed significant DX243 dose effects between the 0.01 and the 0.1 mg/kg DX243 treated group, the latter one even reaching ASSRs no longer significantly different to the Sham+Veh control group (Supplementary Figure S5A; Supplementary Table S2).

To further specify this tendency of a protective effect of DX243 for phase-locked auditory responses, we considered that in particular the spontaneous LFPs (the brain oscillation potentials independent of external stimulation) were enhanced in situations of disturbed synchronous stimulus-onset responses, as previously shown in mouse mutants (Marchetta et al., 2024). However, 2 weeks after exposure, no difference in the spontaneous LFP activity in the 4 brain regions between the Sham+Veh control and AT treated groups was observed, with or without DX243 treatment (Supplementary Figure S5B), or after 6 weeks wash-out following the last DX243 injection (not shown).

**FIGURE 4:**
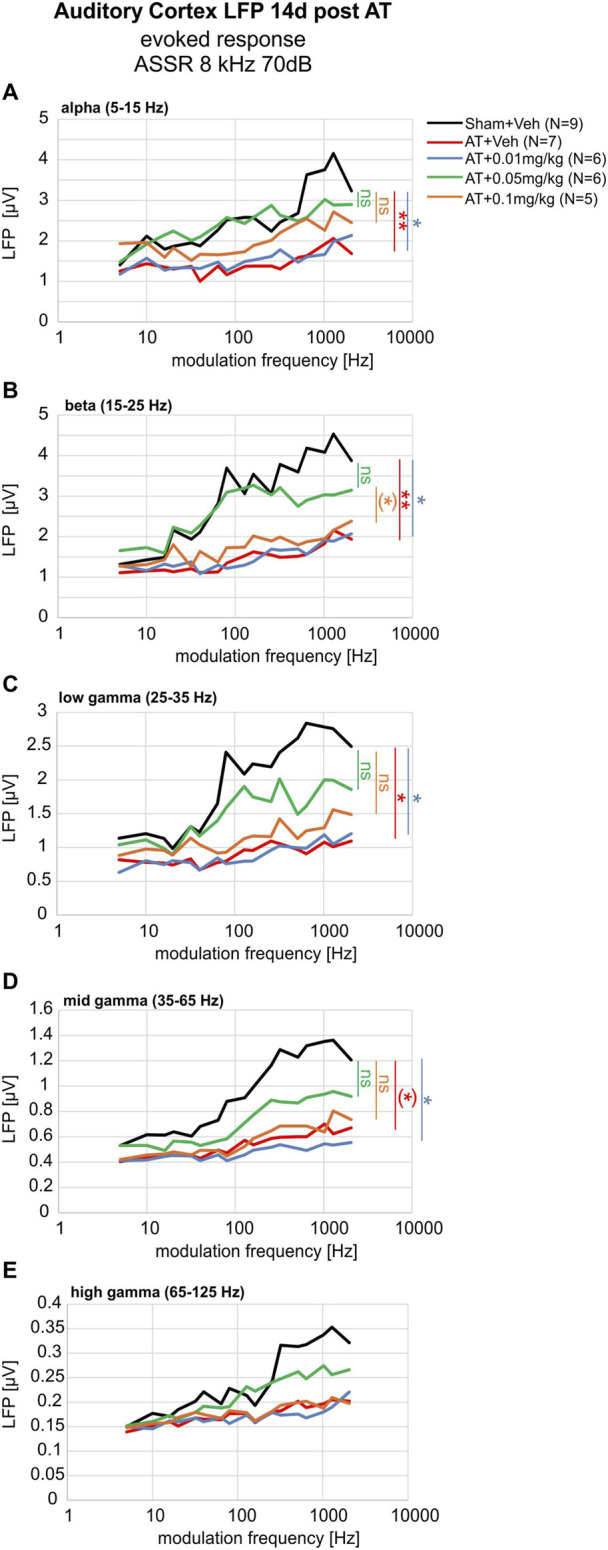
Evoked LFP activity for alpha **(A)**, beta **(B)**, and low-**(C)**, mid- **(D)**, high- **(E)** gamma bands in the auditory cortex in response to an amplitude-modulated stimulus of 8 kHz at 70 dB SPL. The effect on **(A)** alpha (5–15 Hz), **(B)** beta (15–25 Hz), **(C)** low-gamma (25–35 Hz), **(D)** mid-gamma (35–65 Hz), and **(E)** high-gamma oscillations (65–125 Hz) were analyzed by statistically comparing the different treatment groups to the Sham+Veh group (colored asterisks represent Two-way repeated measurement ANOVA results). Evoked oscillations in AT+Veh (red) and AT+0.01 mg/kg DX243 (blue) were significantly smaller for alpha **(A)**, beta **(B)**, low- **(C)**, and mid-gamma **(D)** bands. For the alpha **(A)**, and beta **(B)** oscillatory bands, AT+0.05 mg/kg DX243 (green) almost reached control levels (Sham+Veh, black). Mean of N = 5 - 9 rats. Error bars omitted to maintain clarity. Not quite significant 0.05 < p < 0.1.

To obtain more insight into the target region of the observed DX243 effect on LFP activity, we inspected the LFPs in response to amplitude-modulated tones separately for distinct frequency ranges from alpha to gamma bands (Figure 4). Only recently, cortical envelope-following-responses to amplitude modulation above 80 Hz (mf) were shown to gradually decline up to mfs of 1,000 Hz (Chen and Jennings, 2022), similar to the ASSR responses we show here in the rat (Supplementary Figure S5).

We observed that over a wide range of LFP alpha to gamma bands in controls, the LFP activity gradually increased with increasing modulation frequencies >80 Hz (Figures 4A–E, black line; Supplementary Table S2). After AT (Figures 4A–E, red, n = 7; Supplementary Table S2) and AT+0.01 mg/kg DX243 treatment (Figures 4A–E, blue, n = 6; Supplementary Table S2), we observed a significant decline of LFP activity within all LFP bands. It is remarkable that DX243 0.05 mg/kg and DX243 0.1 mg/kg treatments recovered the LFP responses within alpha, beta, low-, and mid-gamma bands to levels statistically non-significantly different to the Sham+Veh group; this was more so for the DX243 0.05 mg/kg (Figures 4A–C, green; Supplementary Table S2) than for the DX243 0.1 mg/kg treated group (Figures 4A–C, orange; Supplementary Table S2). This challenges the hypothesis that the DX243 effect on LFP activity in response to amplitude-modulated tones reflects a site of action that is peripheral, possibly at a level as early as the auditory nerve.

We conclude from these findings that (i) the daily injection of DX243 administration for 2 weeks, in a dose-dependent manner, prevented an AT-induced impairment of LFP responses, particularly within the alpha and beta brain-oscillatory bands of EEG recordings. Moreover, DX243 counteracted an AT-induced impairment of LFP responses within a wider brain oscillation spectrum in response to tones amplitude-modulated with frequencies above 80 Hz, when an 8 kHz carrier tone was used. This may suggest an effect of DX243 on more slowly adapting, phase-locked temporal envelope coding. (ii) We also conclude from these findings that this DX243 effect was not related to an effect on spontaneous LFP activities, and thus baseline thalamocortical input activity.

### DX243 protects against auditory trauma-induced loss of noise-sensitive auditory fiber processing

To specify a possible effect of DX243 on temporal envelope coding, we considered that peripheral and cortical auditory processing in masking noise critically relies on temporal envelope coding (Kurioka and Mizutari, 2024; Resnik and Polley, 2021; Oxenham, 2018). To test auditory processing in noise, we recorded auditory brainstem responses to an 8 kHz tone within a spectrally notched masking noise (Supplementary Figures S6A,B), the same stimulation frequency as used for the ASSR carrier (Supplementary Figure S5) and amplitude-modulated, tone-evoked LFP (Figure 4). The ABR response was recorded for 25 ms in the absence (Supplementary Figure S6C) and presence (Supplementary Figure S6D) of the masking noise, with sound level increasing in 3 dB steps. When the 8 kHz tone was presented in the notched-noise masking condition, the growth function of the ABR amplitude was strongly reduced (Supplementary Figure S6E). Depending on the stimulus level, the notched masking noise reduced the ABR amplitude by 54%–59% for higher stimulus levels of 60–99 dB SPL (Supplementary Figure S6E, rectangle), and by 2%–7% for close-to-threshold stimulus levels below 48 dB SPL (Supplementary Figure S6E, arrow). As expected, acoustic trauma also significantly reduced the summed auditory nerve response to the 8 kHz tone (Supplementary Figure S6F, solid red line). When the 8 kHz tone was presented in notched-noise after acoustic trauma, however, the response still further declined (Supplementary Figure S6F, dotted red line), but by much smaller amounts than before acoustic trauma (18%–32% for stimulus levels 60–99 dB SPL and ca. 0% at close-threshold stimulus levels). We conclude that the acoustic trauma conditions dramatically reduced the number of auditory fibers that were the most vulnerable to such trauma, i.e., the lower-spontaneous firing rate fibers (Kujawa and Liberman, 2009). Being not masked by noise - as the high-spontaneous firing rate fibers likely are (Bharadwaj et al., 2019), these fiber types mainly contribute to the elevated growth function of the response to the 8 kHz tone at increasing stimulus levels above 40 dB SPL (Supplementary Figure S6F, rectangle). The damage to (low-SR) auditory nerve fiber processing by acoustic trauma was, however, not entirely complete under our conditions, as after AT, the auditory responses still increased with higher stimulus levels despite background noise (Supplementary Figure S6F, dotted red line).

**FIGURE 5:**
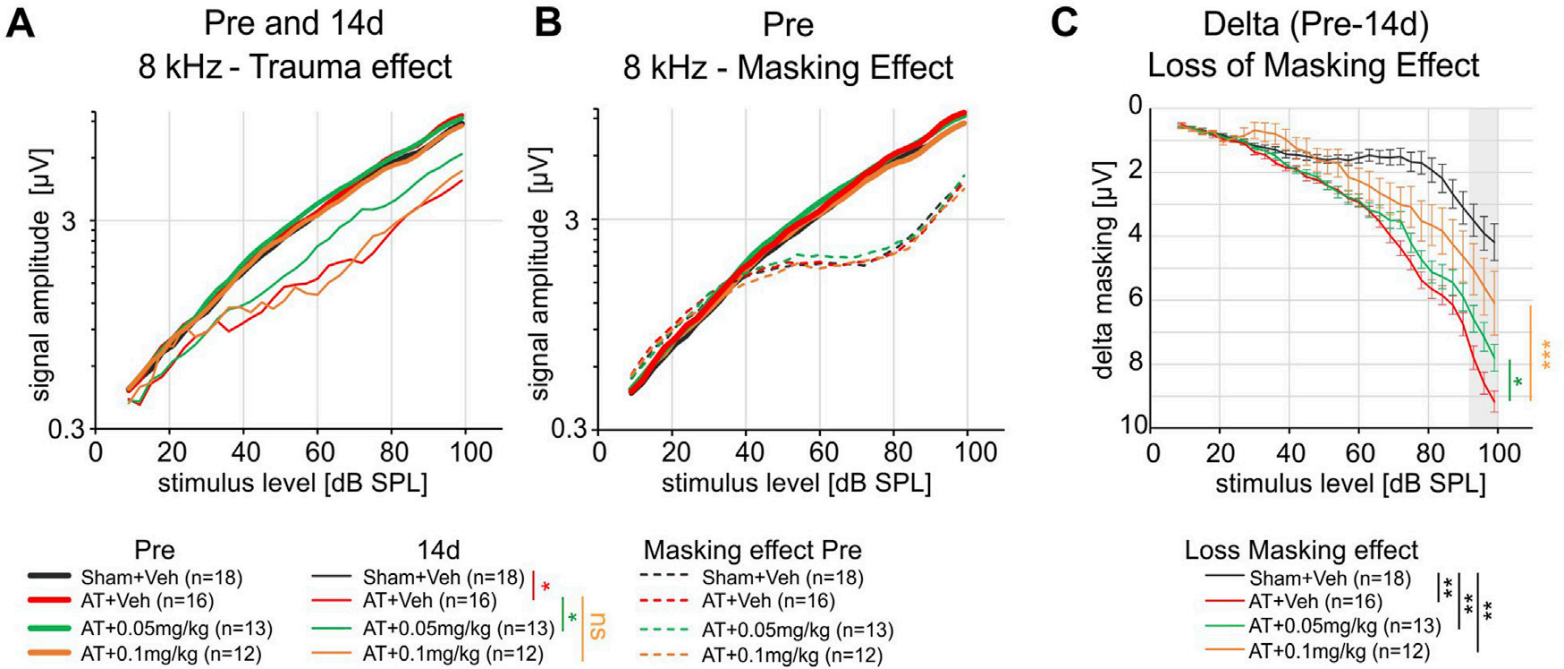
Suppression of the ABR response amplitude to an 8 kHz pure tone by the spectrally notched-noise masker. **(A)** 8 kHz ABR amplitude (peak-to-peak potential within a 4 ms time window) for increasing stimulus levels (dB SPL) pre (thick lines) and 14d post AT (thin lines). 14d after exposure a significant reduction was observed for Sham+Veh vs. AT+Veh groups (Sidak’s multiple comparison test at and above 54 dB SPL, p = 0.0111, asterisk and red vertical bar in 14d legend underneath). For AT+Veh vs. AT+0.05 mg/kg DX243, Sidak’s test at and above 93 dB SPL (p = 0.0168, in green). AT+Veh vs. AT+0.1 mg/kg DX243, Sidak’s n.s. at all stimulus levels (in orange). **(B)** 8 kHz ABR response amplitudes prior to DX243 or vehicle treatment and traumatic sound exposure (pre) to increasing stimulus levels (dB SPL) with (thin dashed lines) and without masking notched-noise (thick lines). The suppressing effect of the masking notched-noise manifests as the stagnation and drop in the growth of the curve above ∼40 dB SPL stimulus level. The masking effect suggests the contribution of a class of auditory fibers sensitive in the higher sound level range to the ABR signal amplitude. **(C)** 14d after acoustic trauma, the ABR signal amplitude dropped for stimulus levels above ca. 30 dB SPL (A, thin lines, see also Supplementary Figure S6) and the suppressing effect of the masking noise was greatly reduced (higher delta masking noise, µV values) compared to before trauma and sham exposed controls (black line). DX243 treatment with 0.05 and 0.1 mg/kg reduced the loss of ABR suppression by the masking noise (green + orange lines closer to black than red line), strongly suggesting that DX243 partly conserved the class of fibers sensitive in the higher sound level range. All Two-way ANOVA comparisons showed highly significant differences (p < 0.0001) for the treatment effect. Sidak’s multiple comparison test reached statistical significance between the AT+Veh vs. AT+0.05 mg/kg DX243 (p = 0.0153) and AT+Veh vs. AT+0.1 mg/kg DX243 (p = 0.0002) for sound levels above 90 dB SPL (in figure, grey shaded area). All AT treated groups showed significant loss of masking effect compared to Sham+Veh as indicated by black lines and asterisks in the legend below for sound levels above 60 dB SPL.

When the ABR responses to an 8 kHz tone in noise were tested with different dosages of DX243 (Figure 5), 0.05 mg/kg DX243 was observed to significantly mitigate the AT-induced drop of growth response (Figure 5A: Sham+Veh vs. AT+Veh: Two-way ANOVA factor treatment p < 0.0001, Sidak’s multiple comparison test p < 0.05 at and above 54 dB SPL. AT+Veh vs. AT+0.05 mg/kg DX243: Two-way ANOVA p < 0.0001, Sidak’s p < 0.05 at and above 93 dB SPL. AT+Veh vs. AT+0.1 mg/kg DX243: Two-way ANOVA p = 0.014, Sidak’s n.s. at all stimulus levels; Supplementary Table S2). This indicates that DX243 preserved peripheral response amplitude to 8 kHz tones at higher sound levels. ABR amplitudes (peak-to-peak potential within a 4 ms time window) were tested using increasing stimulus levels (dB SPL) with (thin lines, masked) and without masking notched-noise (thick lines) before traumatic sound exposure or vehicle treatment (Figure 5B; Supplementary Table S2).

The suppressing effect of the masking notched-noise manifested itself as the stagnation and fall in the growth of the curve achieved above approx. 40 dB SPL stimulus level. The masking effect suggests the contribution of a class of auditory fibers sensitive in the higher sound-level range to the ABR signal amplitude; these are likely low-SR auditory fibers with high activation thresholds. Importantly, the number of auditory fibers that contributed to the 8 kHz response in notched-noise in the AT condition was significantly less reduced 2 weeks after AT both in the presence of 0.05 mg/kg DX243, and of 0.1 mg/kg DX243, as demonstrated when only the difference (delta) of the response to the 8 kHz auditory stimulus between conditions with and without notched-noise masking was calculated (Figure 5C, orange, green, grey shaded area; Supplementary Table S2).

In summary, this finding demonstrates that at 0.05 mg/kg and 0.1 mg/kg dosages, DX243 preserved an AT-induced drop of auditory growth function in the absence and presence of notched-noise, particularly for auditory responses at higher sound levels.

### DX243 protects auditory trauma-induced loss of cochlear neuropathy

**FIGURE 6:**
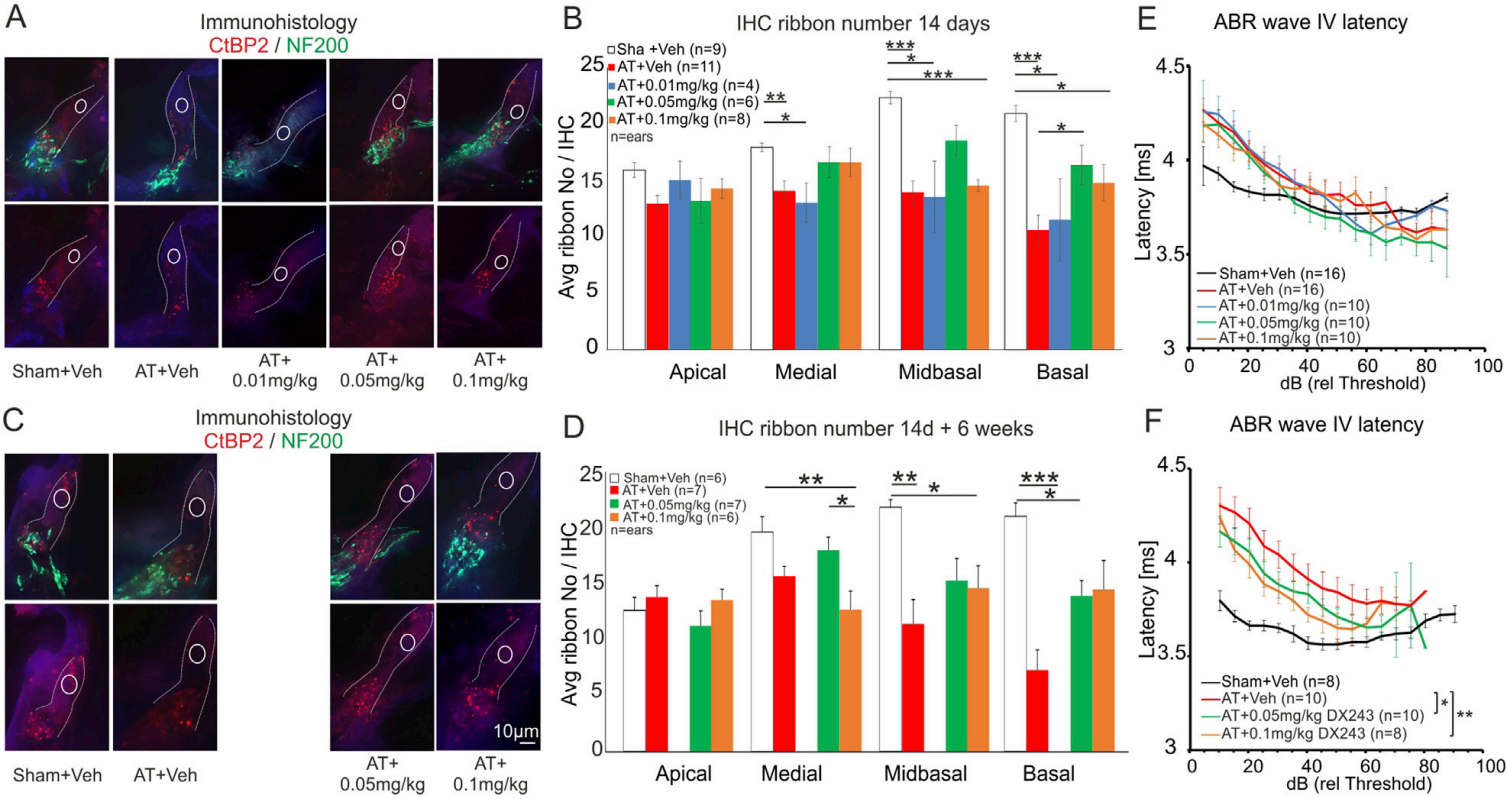
Peripheral damage seen in IHC ribbon number reduction and ABR wave IV latency increase. IHC ribbon histology 14d post AT **(A,B)** and 14d + 6 weeks **(C,D)** after exposure. **(A,C)** Example of immunohistochemical staining of CtBP2 (red) and NF200 (green) for Sham+Veh, AT+Veh, AT+0.01 (not investigated for C), AT+0.05 and AT+0.1 mg/kg DX243. **(B,D)** Average IHC ribbon numbers for all cochlear turns 14d post AT and 14d + 6 weeks after exposure. **(B)** For medial, midbasal and basal turns a significant reduction in IHC ribbon number is seen between Sham+Veh vs. AT+Veh and AT+0.01 mg/kg DX243 groups. In addition, midbasal and basal AT+0.1 mg/kg DX243 shows significantly reduced ribbon numbers vs. Sham+Veh, too, while the AT+0.05 mg/kg DX243 group does not. For the basal turn ribbon numbers are significantly higher for AT+0.05 mg/kg DX243 vs. AT+Veh. **(D)** For the midbasal and basal turns, a significant reduction in IHC ribbon number in the AT+Veh group is seen 14d + 6 weeks after the end of treatment in comparison to the Sham+Veh group. Although the sound exposed groups treated with DX243 also show a reduced ribbon number compared to Sham+Veh controls, both DX243-treated groups show higher ribbon numbers than the AT+Veh group. **(E)** Wave IV latencies 14d post AT and **(F)** 14d + 6 weeks after exposure. All sound-exposed rats showed prolonged ABR wave IV latencies. A treatment-specific effect was only observed at 14d + 6 weeks. The AT+Veh group shows a significant further prolongation of ABR wave IV latency in comparison to both 0.05 and 0.1 mg/kg DX243 groups (Tukey’s multiple comparison test: p = 0.049 and 0.0041, respectively).

Aiming to investigate whether the DX243-induced preservation of 8 kHz ABR wave amplitudes in the presence and absence of noise was linked to a cochlear synaptopathy, we analyzed the number of the IHC ribbons. IHC ribbons contribute to spontaneous and evoked discharge rates of rodent ANF through their influence on the readily releasable vesicle pool at IHC synapses (Ruttiger et al., 2017; Kujawa and Liberman, 2009). The ribbon structures were stained by antibodies directed against the RIBEYE protein CtBP2. In our hands, CtBP2 was colocalized with the postsynaptic neuronal afferent marker protein NF200 (Figure 6A). As also shown in numerous studies in rodents (Ruttiger et al., 2017; Kujawa and Liberman, 2009), auditory trauma in rats is followed by reduced numbers of CtBP2 positive spots at the base of IHC synapses. This is most pronounced in higher-frequency cochlear turns, as, e.g., shown for the midbasal cochlear turn (Figure 6A, Sham+Veh, AT+Veh). DX243 0.05 mg-treatment reduced the IHC ribbon loss (Figure 6A, compare AT+Veh and AT+0.05 mg/kg DX243, red dots). A quantification of the acoustic trauma-induced reduction of presynaptic CtBP2-positive release sites was performed in rat cochlear sections from all treatment conditions, 2 weeks (Figure 6B) and 8 weeks (Figure 6D) post-AT for all DX243 dosages. A significant reduction of ribbon numbers was most pronounced in midbasal and basal cochlear turns 2 weeks post-AT (Figure 6B; Supplementary Table S2) and 8 weeks post-AT (Figure 6D; Supplementary Table S2), with a maximal protective effect for the AT+0.05 mg/kg DX243 group (Figure 6B, green bar; Supplementary Table S2).

A progression of IHC ribbon loss after AT between 2 and 8 weeks (compare red bar, basal turn Figures 6B,D) was also seen when analyzing the ABR peak latencies of ABR wave IV (Figures 6E,F, red line). ABR peak latencies were calculated as the time of the occurrence of the peaks with reference to the stimulus onset (absolute latencies) (Burkard and Don, 2007). As seen in Figures 6E,F ABR wave IV latency peaks delay further after acoustic trauma between 2 and 8 weeks (Figure 6E,F, red line). DX243 at both 0.05 mg/kg and 0.1 mg/kg DX243 partially slowed this process (Figure 6F, compare red with green or orange line).

In summary, the findings suggest that DX243 can counteract AT-induced peripheral impairments such as IHC ribbon loss, with an identical dose-effect profile as shown for DX243 effects on AT-induced cortical responses.

### DX243 supports auditory processing of rapid sequence acoustic impulses after auditory trauma

We tested whether DX243 can preserve the capacity of IHC synapses to follow a rapid sequence of acoustic stimuli by presenting a succession of impulses with decreasing time intervals. This temporally challenging condition may provide insights into DX243 influences on, e.g., the metabolic supply of the IHC synapses.

**FIGURE 7:**
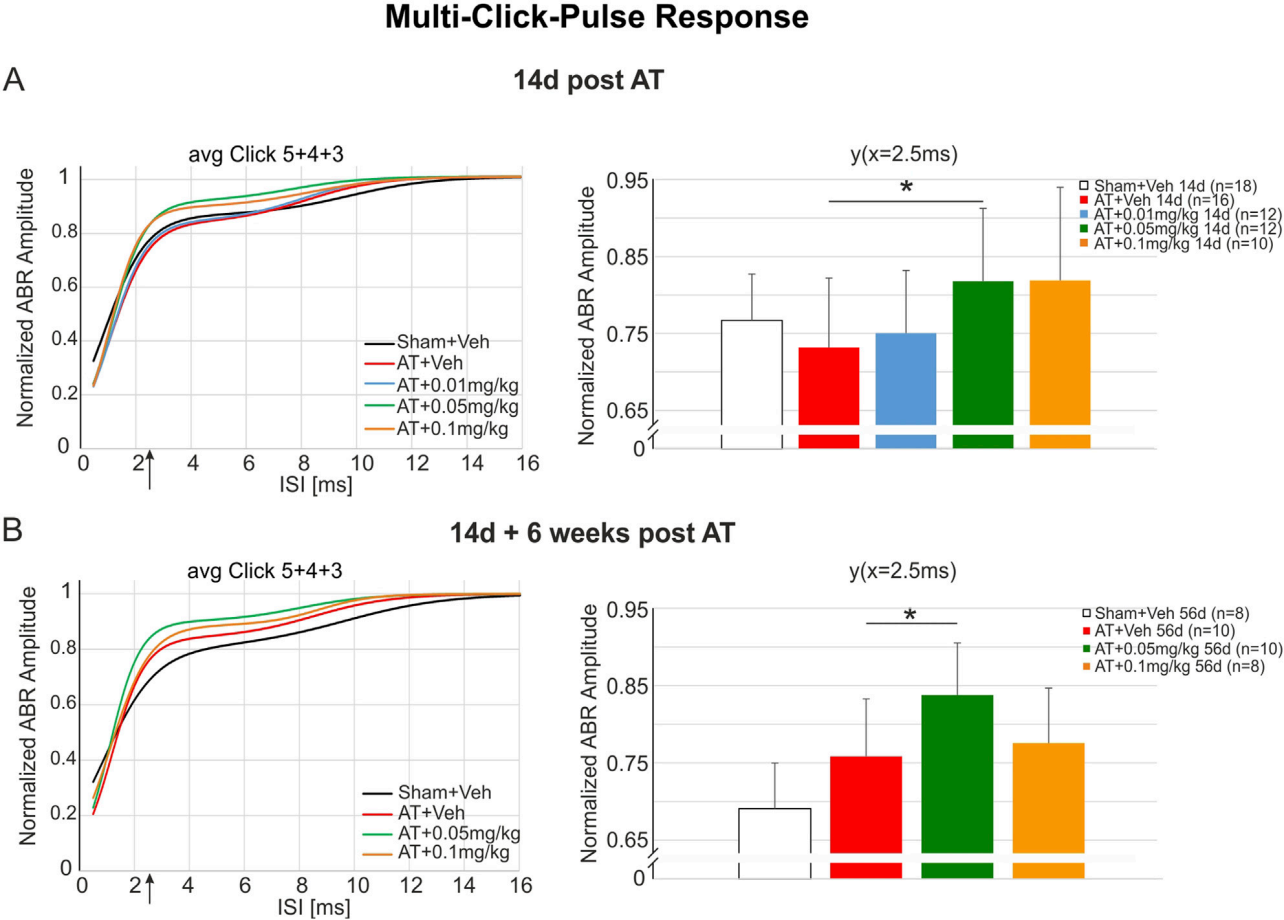
Multi-click responses 14d and 14d + 6 weeks post AT. **(A)** 14d post AT the normalized ABR responses show an elevation of the 0.05 mg/kg and the 0.1 mg/kg DX243-treated groups in comparison either to Sham+Veh or the AT+Veh group [**(A)**, left]. Statistical analysis shows that the fit function at ISI = 2.5 ms of the AT+0.05 mg/kg group is significantly larger compared to the AT+Veh group [**(A)**, right; p = 0.03]. Similar results were found 6 weeks after the end of the 14-day treatment period **(B)**. AT+Veh vs. AT+0.05 mg/kg DX243 [**(B),** right; p = 0.0358]. At both time points after AT (14d and 14d + 6 weeks), the fit curves demonstrate that compared to the Sham+Veh all AT-treated groups show reduced normalized ABR response amplitudes at ISI = 0.5 ms. For details of the fit parameters see Supplementary Table S3.

Acoustically exposed rats with and without DX243 treatment were presented with a series of repeated click stimuli at a stimulus level 30 dB above threshold (SL), with decreasing inter-stimulus time intervals. The ABR responses to these multi-click pulse (MCP) protocols were recorded as described in the methods. Two weeks after sham or traumatic sound exposure and treatment with vehicle or DX243 (Figure 7A). The fit generated from the group means of the individually fitted coefficients for each investigated ear using a double-Boltzmann function demonstrated an elevation of the ABR responses in AT+0.05 mg/kg (green) and AT+0.1 mg/kg groups (orange) that was slightly reduced in the AT+Veh (red) compared to Sham+Veh (black, Figure 7A, left). This observation was confirmed through statistical analysis performed at the fit function at ISI = 2.5 ms, which for the AT+0.05 mg/kg DX243 group demonstrated a significantly larger enhancement compared to the AT+Veh group shown for 2 weeks post AT (Figure 7A, right). Similar results were observed for 14d + 6 weeks (Figure 7B).

In conclusion: The small but remarkable, and statistically significant improvement found in DX243-treated animals for auditory responses responding to rapid, successive acoustic stimuli after auditory trauma, indicates a positive effect of DX243 on IHC synaptic integrity under challenging conditions.

**FIGURE 8:**
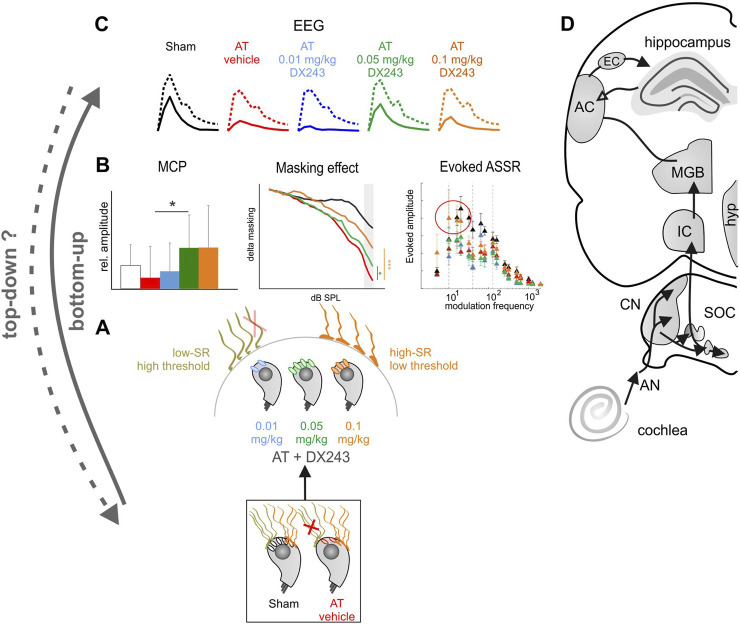
**(A)** The present study suggests a bottom-up effect of DX243 on the preservation of synaptic endings of distinct auditory neural fibers. **(B)** The identical dose-dependence of DX243 on AT-induced cochlear synaptopathy and AT-induced reduction on MCP, Masking effect (Notched-Noise) and evoked ASSR as well as on **(C)** evoked LFP responses recommend DX243 as a drug with **(D)** peripheral and central protective function. Minor effects on induced cortical field potentials in the alpha/beta band (see results) may indicate an intra-cortical effect that necessarily would influence top-down circuits (dotted arrow top-down?). We conclude that DX243 exhibit a positive preservation on exactly those auditory fibers that are most vulnerable to aging and acoustic trauma. (SR) spontaneous firing rate, (AN) Auditory nerve, (CN) Cochlear nucleus, (SOC) Superior olivary complex, (IC) Inferior colliculus, (MGB) Medial geniculate body, (AC) Auditory cortex, (EC) Entorhinal cortex, (hyp) Hypothalamus.

Overall conclusion: In the present study, we demonstrate a preservation of cortical field potentials and ASSR responses after acoustic trauma resulting from daily injections of DX243 for 2 weeks, which persisted for 6 weeks after the last DX243 injection (Graphical abstract, Figure 8). The protective DX243 effect dominated mainly the LFP oscillatory bands of lower frequency (alpha, beta), and was particularly visible for LFP responses if separated for distinct frequency bands in response to amplitude-modulated tones of modulation frequencies above 80 Hz. This indicates a peripheral origin of DX243 LFP effects, as the upper limit of phase-locking in the cortex is predicted to be maximally 40 Hz (Graphical abstract, Figure 8, EEG). Agreeing with this, DX243 changed some features of peripheral temporal envelope coding. Here, DX243 preserved AT-induced attenuation of the growth function of an 8 kHz tone in the absence and presence of notched-noise masking at high stimulus levels (Graphical abstract, Figure 8, masking effect). Finally, DX243 preserved IHC ribbon numbers, possibly dominated by low-SR high-threshold auditory fibers (Graphical abstract, Figure 8, low-SR high-threshold) and the integrity of IHC synapses seen in response to multi-click acoustic stimuli with decreasing interstimulus time intervals (Graphical abstract, Figure 8, MCP). Conclusively, DX243 left auditory processes that rely on proper phase-locked synchronous activity at stimulus onset unaffected (spontaneous LFP, close-to-threshold ASSR, close-to-threshold ABR wave growth function in the absence or presence of masking notched-noise). In contrast, DX243 rather preferentially preserved auditory growth function at higher stimulus levels that are suppressed by AT and masked by noise.

## Discussion

We observed that daily injection of DX243 counteracted an acoustic trauma-induced decline of cortical LFP responses. Here, the evidence is brought that these protective effects of DX243 are likely linked to an influence of DX243 on phase-locked synchronized auditory responses to temporal envelope coding originating at the level of the auditory nerve, while leaving cochlear hair cell survival or phase-locked responses at stimulus onset unaffected.

### DX243 does not influence auditory trauma-induced hearing thresholds shifts

The present findings demonstrated that neither of the DX243 treatment conditions influenced AT-induced impairments of mechano-electrical properties of the outer hair cells (OHCs) (Figure 1) that are suggested to define thresholds of auditory brainstem responses (Dallos and Harris, 1978). This is in line with previous observations that neither of the first-invented steroidal dendrogenin alkaloids, known as α-Hydroxy-6β-[2-(1H-imidazole-4-yl)-ethylamino]-cholestan-3β-ol, called Dendrogenin A (DDA) nor 5α-Hydroxy-6β-[3-(4-aminobutylamino)-propylamino]cholest-7-en-3β-ol, named Dendrogenin B (DDB) (de Medina et al., 2009; Maruyama et al., 2008; Fransson et al., 2010) affected cell or neuronal survival. The dendrogenin derivates were rather suggested to preserve, e.g., electrically-evoked ABR (eABR) thresholds after CI implantation in guinea pigs, similar to GDNF (Fransson et al., 2010) or to other neurotrophic factors (Agterberg et al., 2009; Jørgensen et al., 2012). These trophic factors were predicted to act through pathways influencing neurite outgrowth or dendrite stability rather than neuronal survival. In accordance with this, in these previous experiments, Dendrogenin derivates exhibited no effect on spiral ganglion survival, but only on eABR thresholds. This led to the suggestion that Dendrogenin derivates preserve axonal connectivities and/or promote their growth (Fransson et al., 2015). Such a mechanism, the argument went, would explain improved eABR thresholds through higher numbers and/or better quality of the neural connections with the spiral ganglion neurons, thereby improving cochlear implant efficacy (Fransson et al., 2015). The current finding that DX243 did not affect hearing thresholds after auditory trauma and is unlikely to rescue OHC survival, is thus in line with previous studies (Maruyama et al., 2008; Fransson et al., 2015; Fransson et al., 2010).

### DX243 counteracts auditory trauma-induced reduction of evoked LFP post-AT in the auditory cortex

In the present study, a daily injection of DX243 for 2 weeks after AT was shown to counteract a significant and permanent diminution of sound-evoked LFP activity after acoustic overexposure (Figure 2).

The noise trauma of a 116 dB 10 kHz tone was chosen as these trauma conditions used in rat animal models repeatedly lead to a distinct hearing threshold diminution as well as a defined cochlear synaptopathy mainly in higher frequency regions (Rüttiger et al., 2013; Mohrle et al., 2019; Singer et al., 2018). These mirrored age-related hearing loss and cochlear synaptopathy in the same animal model (Mohrle et al., 2019; Rüttiger et al., 2007). This was crucial to finally relate the observed noise-induced changes in aging, shown to be a lifelong consequence of mild, noise-induced cochlear damage (Wu et al., 2019).

Keeping this in mind, it was interesting to see that the protective DX243 effect lasted 6 weeks after the cessation of DX243 treatment. It was seen particularly in response to lower-frequency acoustic stimuli, up to best frequencies around 10 kHz, which is the frequency range where the hearing of rodents is the most sensitive, and a best-frequency range considered crucial for communication and behavior (Hage and Ehret, 2003; Hernández et al., 2005). Regarding the effect of DX243 on AT-induced LFP within separated frequency bands, it is crucial to reconsider that in the mammalian brain, oscillations and synchronization have been ubiquitously found at the level of single neurons, local neural circuits, and brain-wide networks, from deep brain nuclei to neocortex and across a variety of species (Buzsaki et al., 2013). This has in the meantime also been shown for the auditory system through numerous studies confirming that manipulations of peripheral auditory input – as performed here through acoustic trauma – led to altered neural brain oscillations in humans (Peelle and Wingfield, 2016; Zhao et al., 2024; Allison et al., 1984; Bertoli et al., 2002; Wostmann et al., 2015) and following acoustical, mechanical, or drug-induced trauma in animals (Calford et al., 1993; Norena et al., 2003; Robertson and Irvine, 1989; Yang et al., 2007; Parameshwarappa and Norena, 2024; Resnik and Polley, 2021; Gnaedinger et al., 2019).

In the present study, we demonstrated that auditory trauma profoundly lowered LFP amplitudes within a wide frequency oscillation range (Figure 3; Figure 4). A significant protecting DX243 effects on the AT-induced lowering of LFP activity in response to lower best-frequency tonal stimulation (<11 kHz) was most pronounced for separated LFP frequency bands of alpha, beta, and low-gamma (Figure 3). Strikingly, LFP activity was affected by DX243 over a wider range of LFP frequency bands spanning alpha, beta, low- and higher gamma activity when LFP activity was evoked by amplitude-modulated tones and analyzed for separated frequency bands (Figure 4). While typically, the cortical envelope-following response declined at elevated modulation frequencies >80 Hz, as also here observed for ASSR responses (Supplementary Figure S5A), and as previously shown in humans for envelope-following responses upon scalp electrodes (Chen and Jennings, 2022), we observed enhanced LFP activity to modulation frequencies of tones >80 Hz when LFP was analyzed in separated frequency bands (Figure 4). This is surprising, since the upper frequency limit of phase-locking to the temporal envelope is expected to be appreciably lower in the midbrain and cortex (Joris et al., 2004). In contrast, the synchrony-based modulation transfer function obtained from, e.g., auditory nerve fibers or some neurons in the cochlear nucleus, can exhibit robust neural synchrony over a wide range of modulation frequencies from 10 to 1,000 Hz (Joris et al., 2004). Accordingly, recording of auditory nerve responses through compound action potentials (CAP) from the tympanic membrane in humans in response to amplitude-modulated tones demonstrated increased synchrony to elevated frequencies >80 Hz modulation (Chen and Jennings, 2022). This response pattern mirrors the increased LFP amplitude behavior shown in control animals in response to >80 Hz in all LFP frequency bands in the present study (Figure 4). As LFP amplitudes (Figure 3) and ASSR response amplitudes to the 8 kHz carrier to amplitude-modulated tones (Supplementary Figure S5A) declined in the present study for rats with a maximal peak response to 40 Hz, as shown previously in humans (Chen and Jennings, 2022), we conclude that the LFP responses to >80 Hz modulated tones observed in rats in distinct frequency bands were generated at the level of the auditory nerve.

This is supported by the much more pronounced effect of AT on the increase of the LFP response to amplitude-modulated tones >80 Hz (Figure 4) in comparison to LFP amplitudes to evoked frequency dependent pure tones (Figure 3). In conclusion, the DX243 effect on cortical LFP responses would ultimately reflect a response of DX243 to auditory nerve preservation. In that case, the DX243-induced protective cortical LFP responses in the corresponding frequency bands of enhancing brain oscillation would hypothetically point to improved auditory perception through DX243.

Thus, lower-frequency alpha and beta oscillations are assumed to be involved in global, distributed cognitive processing across brain regions (Fontolan et al., 2014; von Stein and Sarnthein, 2000), including attention (Wostmann et al., 2015; Levin et al., 2017; Klimesch, 2012), precision of predictions (Cope et al., 2017; Sedley et al., 2016), and, e.g., speech-in-noise comprehension (Price et al., 2019). Notably, changes in beta activity in humans were strongly correlated with the slope of listeners’ psychometric identification functions (Bidelman and Dexter, 2015), and if deficient, with lower auditory processing steepness, particularly also in noise (Sivarao et al., 2013). Corresponding to this were enhanced alpha/beta activity changes in rodents that were linked to learning-associated, improved task performance (Gnaedinger et al., 2019). Finally, changes in higher-frequency gamma oscillations point to altered contributions to localized processing within sensory cortices (Fontolan et al., 2014; Giraud and Poeppel, 2012).

Regarding the effect of DX243 on > 80 Hz modulated tones in LFP higher gamma frequencies (Figure 4), we may reconsider that higher gamma oscillations are suggested to be most critically dependent on phase-locked synchronized responses at stimulus onset (Hu et al., 2014; Chen et al., 2017; Sohal et al., 2009). Thus, in the auditory pathway, stimulus-onset coding is predicted to dependent on high-SR auditory-fiber processing (see for a review Huet et al., 2019). Our findings, however, indicate that DX243 spares AT-induced effects on phase-locked synchronized responses at stimulus onset. This can be concluded since DX243 left ASSR responses near-threshold sound levels (Supplementary Figure S5A), that critically dependent on synchronous discharge of auditory neurons (Kuwada et al., 2002; Parthasarathy and Bartlett, 2012; Lu et al., 2022), and thus high-SR ANF (Huet et al., 2018; Huet et al., 2019) unaffected, while low-SR ANF with higher activation thresholds poorly contributed to synchronized phase-locked responses at stimulus onset (Huet et al., 2018). An effect of DX243 on high-SR ANF and phase-locked responses at stimulus onset is moreover counter-indicated by the observation that regardless of DX243 treatment, AT did not change spontaneous neural oscillations measured without stimuli at rest (Supplementary Figure S5B). Accordingly, in the case of stimulus onset changes by DX243, we would expect an elevation of spontaneous LFP activity at rest, caused by an elevation of the operation point of thalamocortical input activity on AC neurons in layer IV and a subsequently altered input activity of layer IV to influence infragranular layer V-VI (output) into supra-granular layers I-III (Liu et al., 2015; de Cheveigne et al., 2013), as shown in previous studies in mice (Marchetta et al., 2024).

We so far conclude that DX243 exerts a protective effect on the trauma-induced reduction of cortical LFP responses that are likely to originate in changes in peripheral processing. Here, however, the findings indicate that it is unlikely that DX243 affects OHC survival or the auditory fiber processing that contributes to rapid phase-locked responses at stimulus onset.

### DX243 counteracts the auditory trauma-induced reduction of evoked LFP post AT, particularly in response to lower-frequency stimuli

According to numerous studies, the damage by acoustic trauma to the cochlea is expected to alter the response of cortical neurons to acoustic stimuli in the affected region (Scholl and Wehr, 2008; Calford, 2002b; Calford, 2002a; Norena et al., 2003). We here, however, observed a preferential preservation function of DX243 on the AT-induced decline of LFPs responses, mainly in lower-frequency regions < CF of 10 kHz (Figure 2), although the tonal audiometry indicated the highest damage to higher-frequency regions > CF of 10 kHz (Figure 1). We also observed a slight but significant affect of DX243 on LFP responses evoked by 23 kHz, indicating a preserving DX243 effect on AT-induced damage in extended high-frequency (EHF) regions (Figure 3). Regarding previous studies in rodents (Marcher-Rorsted et al., 2022) and humans (Schirmer et al., 2024) that observed an impact of explicit hearing loss in EHF regions on lower frequencies relevant for speech (<6 kHz), we cannot exclude a relationship between DX243 effects on LFP amplitudes in lower and EHF regions, as discussed for humans (Schirmer et al., 2024). Here, in subjects with hearing-threshold deficits in EHF regions, the recording of neuroelectrical EEG responses was shown to influence temporal-envelope coding (Dapper et al., 2025). Although the mechanism as to how higher frequency regions, in particular EHF regions, may influence lower frequencies, is still speculative (Marcher-Rorsted et al., 2022; Schirmer et al., 2024; Dapper et al., 2025), a possible influence of IHCs synaptic weakness specifically influencing onset coding in EHF regions (Schirmer et al., 2024; Dapper et al., 2025) that may influence temporal envelope coding in lower frequency ranges (Schirmer et al., 2024; Dapper et al., 2025), as already discussed, may be worthwhile to consider in future translational studies of DX243.

We so far conclude that DX243 has a protective effect on those AT-induced impairments of central neural brain oscillation that neither have their origin in OHC loss, nor in critical impairments of phase-locked synchronous responses in the ascending pathway, but nevertheless may impact lower-frequency ranges that in humans play a critical role in speech comprehension.

### DX243 counteracts auditory trauma-induced reduction of the peripheral processes involved in temporal envelope coding

The observations so far have suggested that DX243 has no effect on cortical responses that depend on phase-locked synchronous responses at stimulus onset. The synchronous firing rate at the onset of the stimulus is a feature that critically depends upon the sensitivity of high-spontaneous firing rate (high-SR), low-threshold ANFs, which define latencies and perception thresholds (Rhode and Smith, 1986; Zohar et al., 2011; Meddis, 2006; Heil et al., 2008) and defines temporal fine-structure coding below phase-locking limits (Huet et al., 2019). In contrast, increasing sound-pressure levels gradually recruits auditory fibers with lower-spontaneous firing rates and higher activation thresholds that contribute little to the synchronization of ANFs (Huet et al., 2019).

The so-called ‘hidden hearing loss’ refers specifically to the damage to peripheral synaptic processes of the low-SR, high-threshold ANFs (Liberman and Kujawa, 2017), which are said to play a role in coding supra-threshold sound features of speech in noise (Monaghan et al., 2020; Liberman and Kujawa, 2017; Plack et al., 2014; Wu et al., 2019; Chambers et al., 2016; Bakay et al., 2018; Hesse et al., 2016; Keithley, 2020; Gomez-Alvarez et al., 2023; Kujawa and Liberman, 2019; Chen et al., 2021). Thus, subjects with hidden hearing loss, and with speech-comprehension deficits in noise, display normal pure-tone and speech-audiometry thresholds in quiet, and well-synchronized ABRs (Chen, 2018; De Siati et al., 2020).

Based on several observations, we argue here that DX243 preferentially effects low-SR ANF that have higher activation thresholds: (i) the ABR threshold shifts, as well as (ii) the ABR wave I amplitude were not affected, while (iii) DX243 protected the 8 kHz tone-induced growth function in the absence and presence of background noise (Figure 5). This is discussed in the following in more detail: In the high-frequency range (above phase-locking limits), high-SR fibers with low activation thresholds reach their maximum discharge in noise, making them inefficient in responding to tones, and only the low- and medium-SR that have more elevated thresholds can encode tones in noise (Huet et al., 2019). This suggests that the DX243 effects, mainly seen on the 8 kHz growth function at higher sound levels, have their rational in the larger dynamic ranges of low-SR ANF fibers that respond with increasing firing rates only at higher activation thresholds (Schalk and Sachs, 1980). As a result, in the presence of an acoustic stimulus in a continuous noise stimulus (see Supplementary Figure S6, shown for the 8 kHz tone), the low-SR fibers would be less masked, and are thus responsible for the remaining growth function (Supplementary Figure S6). Importantly, these remaining growth functions to an 8 kHz tone in response to higher sound levels are preferentially restored in the presence of DX243 (Figure 5), arguing for DX243 effects on low-SR ANFs.

Low-SR ANF are also the type of auditory fiber that exhibits the highest vulnerability to acoustic trauma and aging, which is likely related to their low levels of mitochondria (Liberman and Kujawa, 2017), providing a rational for its preferential loss following AT in the present study (Supplementary Figure S6F). Although previous studies pointed out that in noise-exposed mice, noise exposure affect both high-SR ANF and low-SR ANF as, e.g., (Suthakar and Liberman, 2021), we nevertheless conclude from the following notched-noise study that there is a preferential effect on those likely low-SR ANF fibers that are not masked by noise (Bharadwaj et al., 2014). Thus, our findings importantly demonstrate that the ABR response to 8 kHz tones in the presence of noise is preserved by 0.05 mg/kg DX243 and 0.1 mg/kg DX243 (Figure 5). This occurs particularly for higher sound levels that were thus seen to be less susceptible to the masking noise level (>60 dB SPL). We therefore suggest that DX243 exhibits a capacity to preferentially preserve and sustain those auditory fibers that, due to their wide dynamic range to higher intensity sound, especially contribute to hearing in background noise (Bharadwaj et al., 2019; Monaghan et al., 2020; Kujawa and Liberman, 2019). The preservation of this most vulnerable auditory fiber type by DX243 is of particular interest. Thus, speech comprehension in noise and with age is currently assumed to be compromised as a result of age- and acoustic-noise damage that induce impairments of peripheral synaptic endings, preferentially on low-SR ANF (Monaghan et al., 2020; Liberman and Kujawa, 2017; Plack et al., 2014; Frisina, 2009; Wu et al., 2019; Füllgrabe et al., 2014). This is predicted to occur despite normal pure-tone and speech audiometry thresholds in quiet, and well-synchronized ABRs (Kraus and White-Schwoch, 2017; Chen, 2018). The diminished low-SR ANF processing is hypothesized to lead to speech-comprehension deficits, due to a disadvantage in differentiating formant contrasts above phase-locking limits, as previously shown in human studies (Schirmer et al., 2024).

## In conclusion

In line with the previous assumption of a protective effect of dendrogenin on preserving the functional efficacy of cochlear afferent neurites following cochlear implants, rather than in spiral ganglion neuron survival (Fransson et al., 2015), the present study suggests an effect of DX243 on the preservation of synaptic endings of distinct auditory neural fibers (Figure 8). The identical dose-dependence of DX243 on AT-induced cochlear synaptopathy and AT-induced reduction of evoked LFP responses recommend DX243 as a drug with peripheral/central protective function. The evidence for a positive preservation of exactly those auditory fibers that are most vulnerable to aging and acoustic trauma, contributing to hearing in background noise is here provided on various levels. A pharmacological intervention at an early stage would be of great interest, as cochlear synaptopathy due to accumulating acoustic injuries increases over time, leading to permanent changes in the auditory pathway (Viana et al., 2015; Wu et al., 2021; Fetoni et al., 2015). As hearing loss can be compensated over a relatively long time (Viana et al., 2015; Wu et al., 2019; Wu et al., 2021) a therapeutic window for DX243 treatment with age (counteracting accumulating acoustic injuries) exists, in which pharmacological prevention could be applied.

## Data Availability

The raw data supporting the conclusions of this article will be made available by the authors, without undue reservation.
