# Supplementary figures and images for: DX243 counteracts both acoustic trauma-induced reduction in cortical brain oscillations and cochlear synaptopathy
Source: Front Pharmacol. 2026 Jan 14;16:1673189. doi: 10.3389/fphar.2025.1673189 (PMC12847371; doi:10.3389/fphar.2025.1673189)

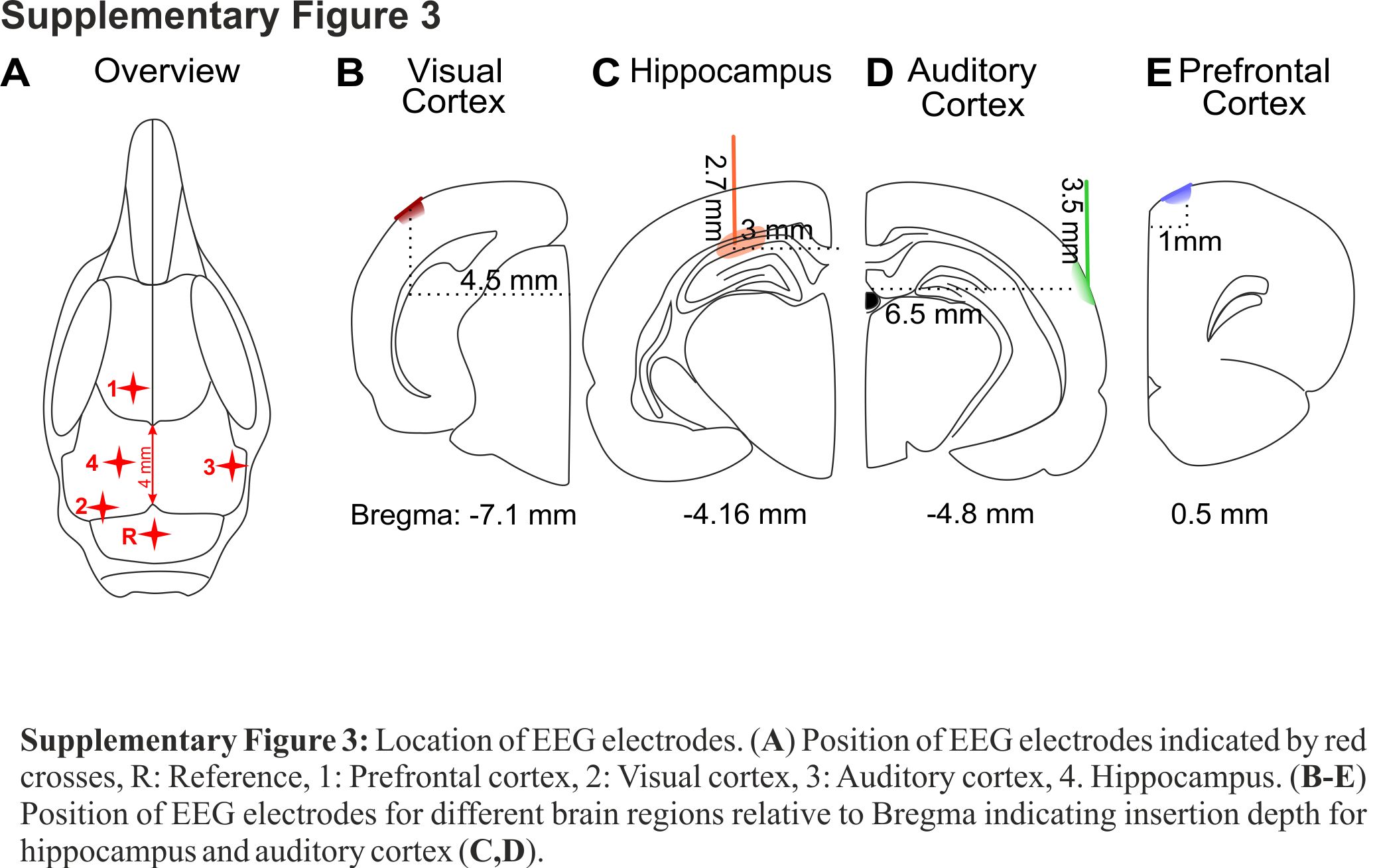

Supplement: Supplementary file 1 [file Image3.jpeg]

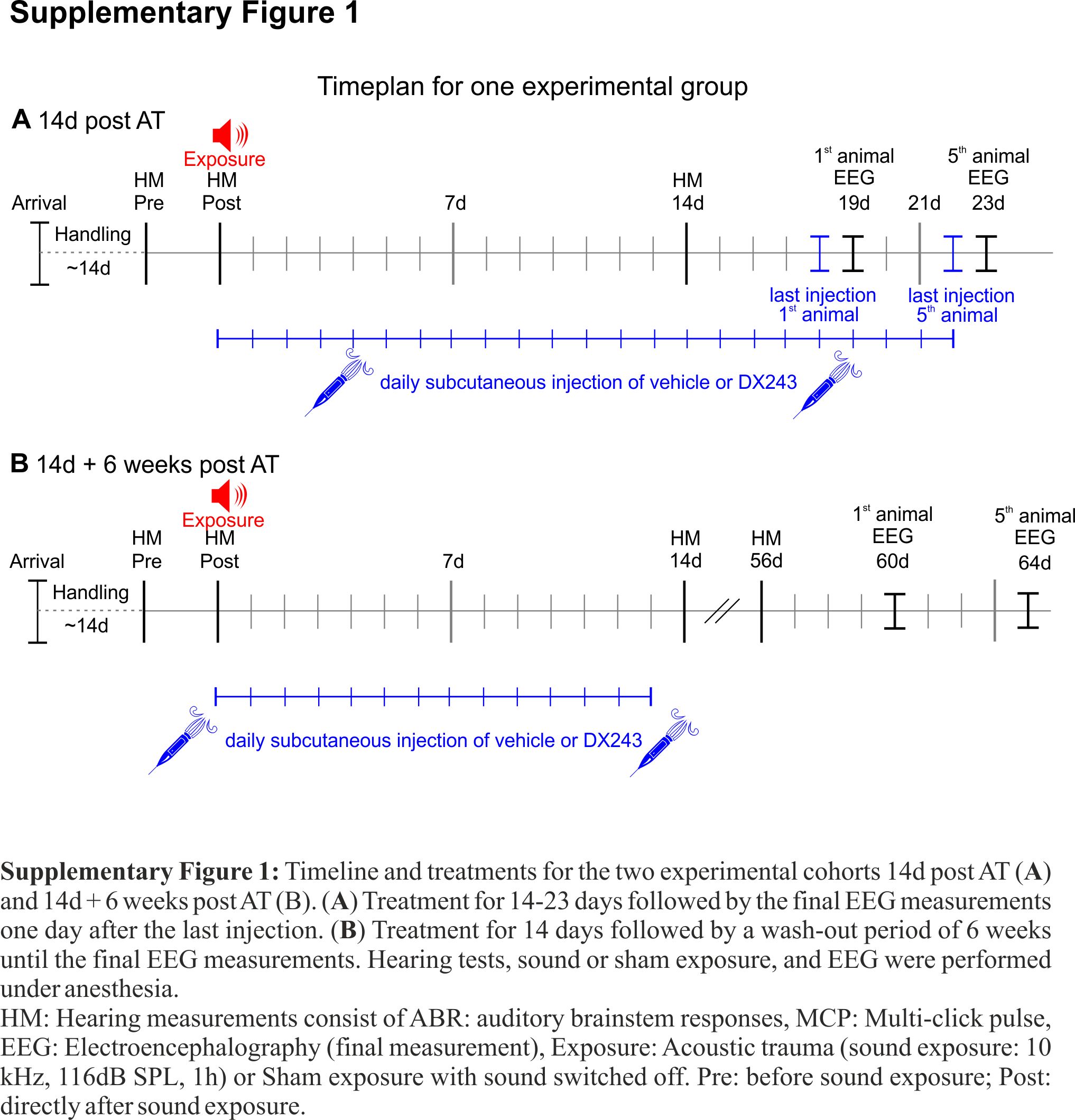

Supplement: Supplementary file 3 [file Image1.jpeg]

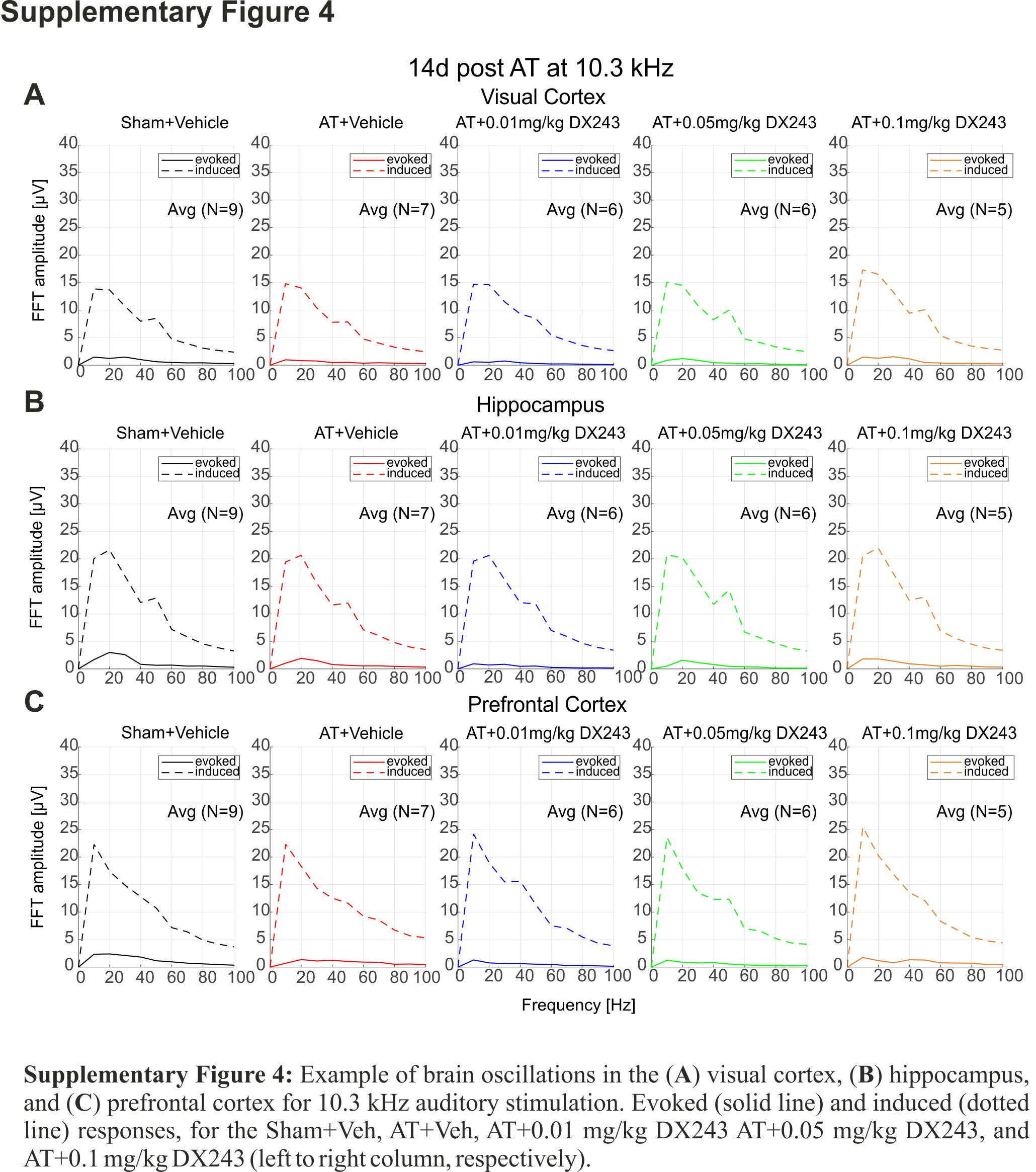

Supplement: Supplementary file 4 [file Image4.jpeg]

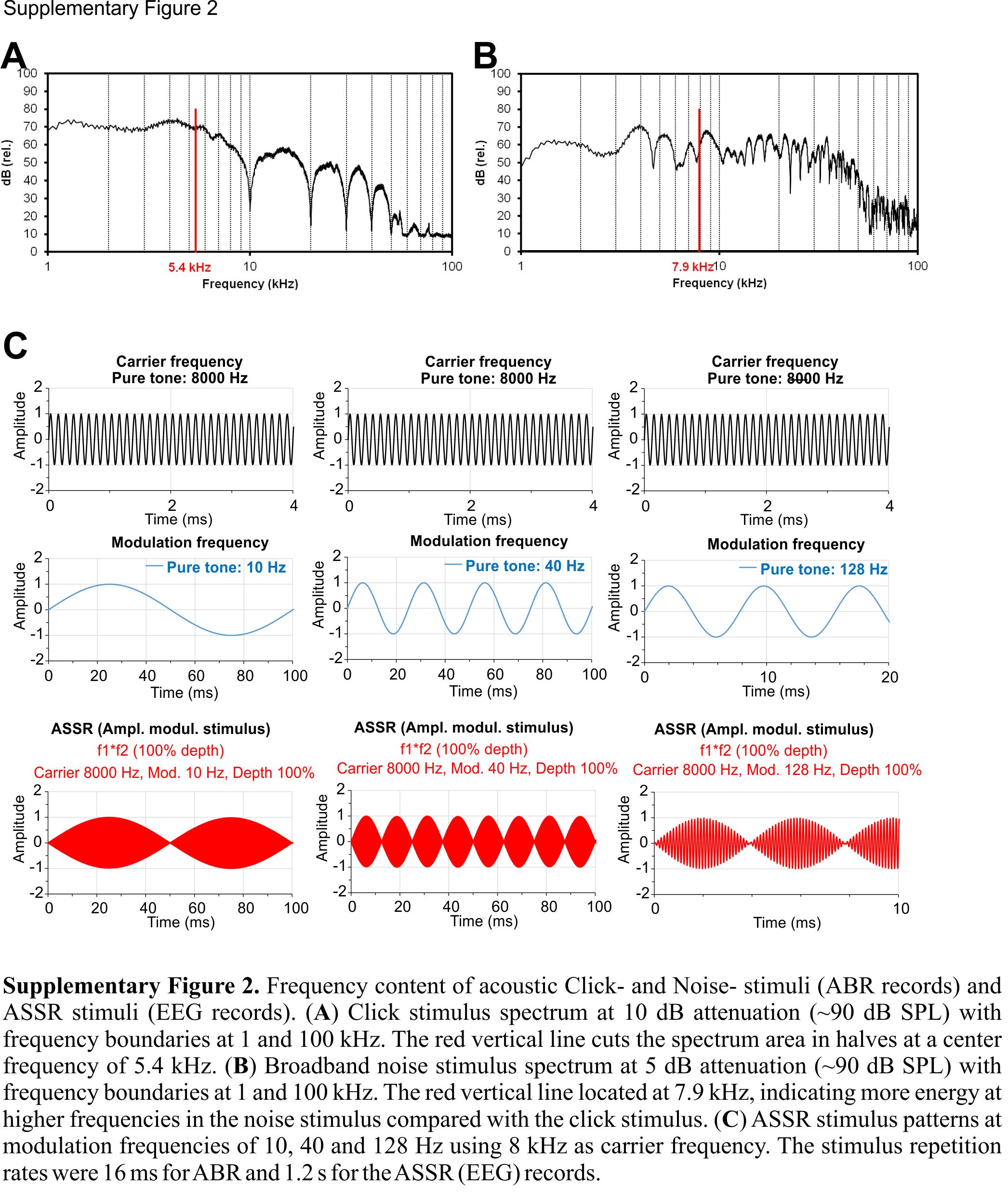

Supplement: Supplementary file 5 [file Image2.jpeg]

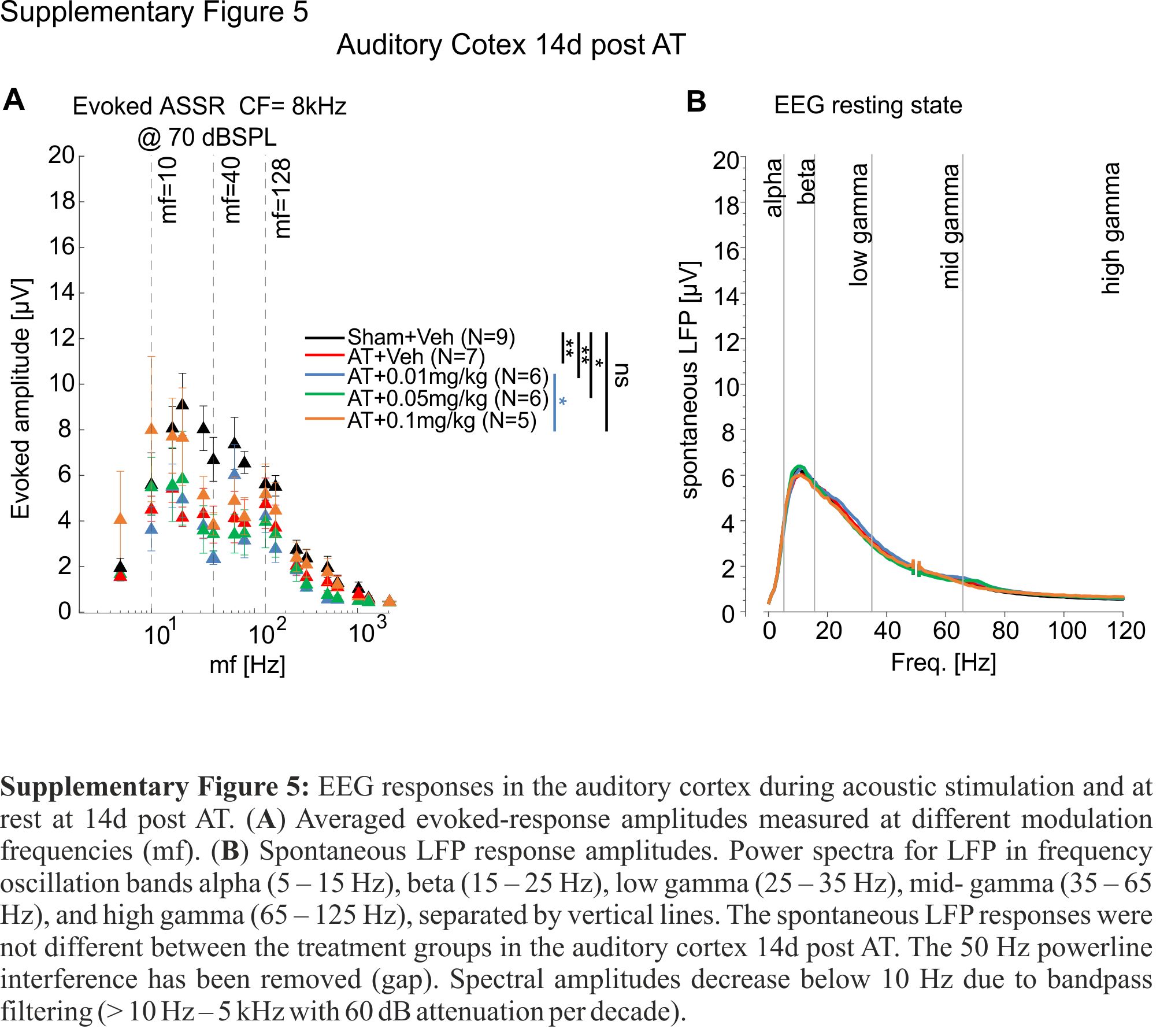

Supplement: Supplementary file 6 [file Image5.jpeg]

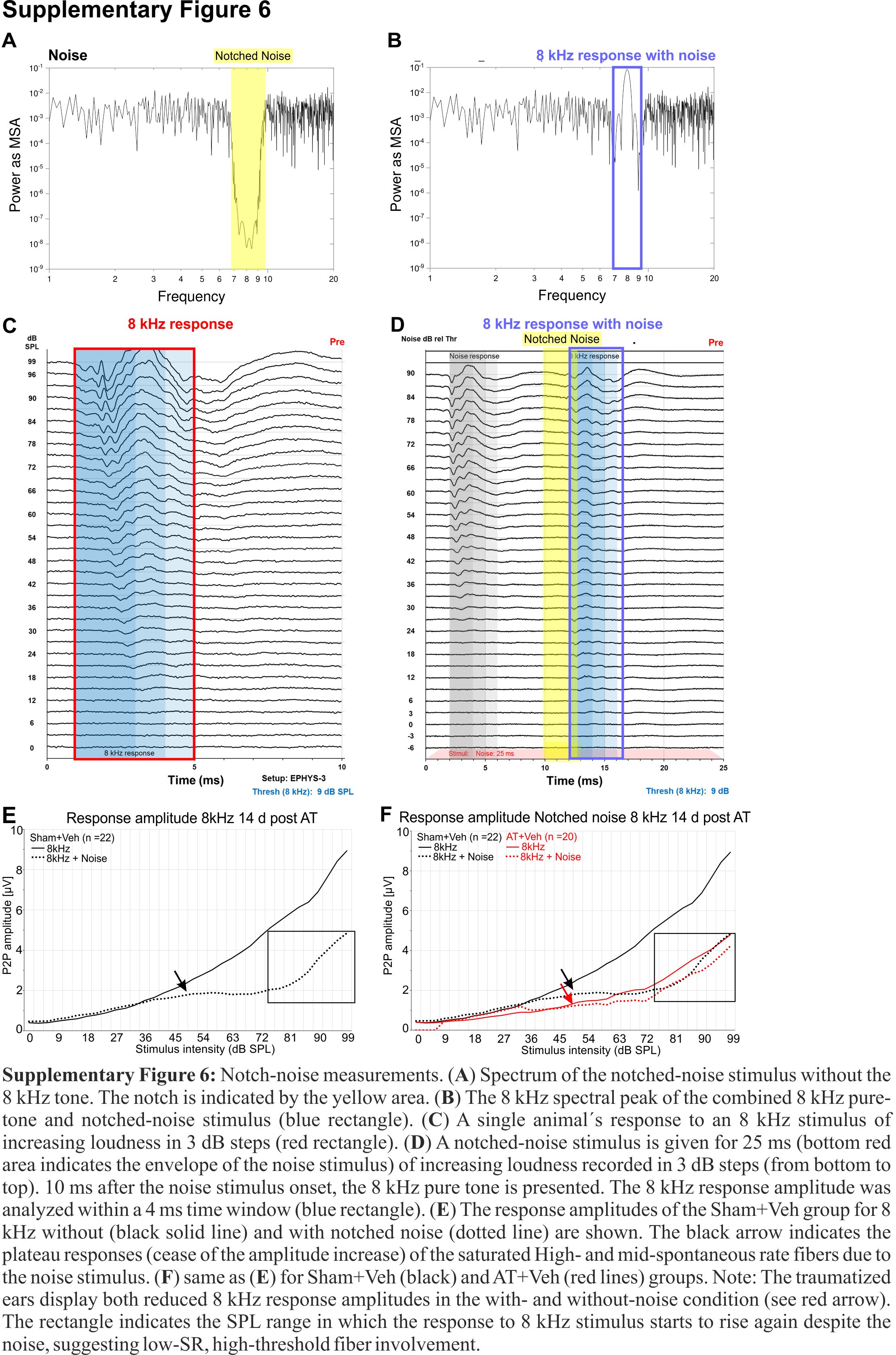

Supplement: Supplementary file 9 [file Image6.jpeg]
