# Supplementary material for: DX243 counteracts both acoustic trauma-induced reduction in cortical brain oscillations and cochlear synaptopathy
Source: Front Pharmacol. 2026 Jan 14;16:1673189. doi: 10.3389/fphar.2025.1673189 (PMC12847371; doi:10.3389/fphar.2025.1673189)
Supplement: Supplementary file 2 [file Table1.docx]

**Supplementary Table 1:** All animals (N=53) used in this study for ABR & EEG records, listed in chronological order for all sets of experiments for both cohorts and all five groups of treatment. * not used for EEG

| **Cohort 1** |  | **14d s.c.** |  |  |  |
| --- | --- | --- | --- | --- | --- |
|  | **Sham+Veh** | **AT+Veh** | **AT+0.01mg/kg** | **AT+0.05mg/kg** | **AT+0.1mg/kg** |
| **Block 9** | DXR150 | DXR152 |  |  | DXR151 |
|  | DXR154 | DXR153* |  |  | DXR155 |
|  |  | DXR156 |  |  | DXR157 |
| **Block 10a** | DXR158 | DXR160 |  | DXR161 | DXR159 |
| **Block 11a** | DXR170 | DXR171 |  | DXR167 | DXR169 |
| **Block 13** | DXR187 | DXR188 | DXR189 | DXR190 |  |
|  | DXR191 | DXR192* | DXR194 | DXR196 |  |
|  | DXR193 |  | DXR195 |  |  |
| **Block 14** | DXR199 | DXR197 | DXR200 | DXR198 |  |
| **Block 15** | DXR202 | DXR204 | DXR203 | DXR201 |  |
|  |  |  | DXR205 |  |  |
| **N = 35** | 9 | 9 (7) | 6 | 6 | 5 |
|  |  |  |  |  |  |
| **Cohort 2** |  | **14d s.c. + 6 wks** | |  |  |
|  | **Sham+Veh** | **AT+Veh** | **AT+0.01mg/kg** | **AT+0.05mg/kg** | **AT+0.1mg/kg** |
| **Block 10b** | DXR163* | DXR165 |  | DXR166 | DXR164 |
| **Block 11b** | DXR176 | DXR172 |  | DXR175 | DXR174 |
| **Block 12** | DXR181* | DXR179 |  | DXR177 | DXR178 |
|  | DXR186 | DXR184 |  | DXR180 | DXR182 |
|  |  | DXR185 |  | DXR183 |  |
| **N = 18** | 4 (2) | 5 | 0 | 5 | 4 |
