# Supplementary material for: DX243 counteracts both acoustic trauma-induced reduction in cortical brain oscillations and cochlear synaptopathy
Source: Front Pharmacol. 2026 Jan 14;16:1673189. doi: 10.3389/fphar.2025.1673189 (PMC12847371; doi:10.3389/fphar.2025.1673189)
Supplement: Supplementary file 7 [file Table2.docx]

**Supplementary Table 2 (Statistics)**

|  |  |  |  | **Kruskal-Wallis** | **Dunn’s multiple comparison** | **Groups Compared** |  |
| --- | --- | --- | --- | --- | --- | --- | --- |
| **14d post AT** |  | **Click** |  | P<0.0001 **** | P < 0001 **** | Sh to AT |  |
| **(Figure 1A)** |  |  |  |  | P < 0001 **** | Sh to AT+0.01 |  |
|  |  |  |  |  | P < 0001 **** | Sh to AT+0.05 |  |
|  |  |  |  |  | P = 0.0246 * | Sh vs. AT+0.1 |  |
|  |  |  |  | P =0.4871 (n.s.) | n.s. | AT to AT+0.01 |  |
|  |  |  |  |  |  | AT to AT+0.05 |  |
|  |  |  |  |  |  | AT to AT+0.1 |  |
|  |  |  |  |  |  | AT+0.05 to AT+0.1 |  |
|  |  |  |  |  |  | AT+0.01 to AT+0.05 |  |
|  |  |  |  |  |  | AT+0.01 to AT+0.1 |  |
|  |  | **Noise** |  | P<0.0001 **** | P < 0001 **** | Sh to AT |  |
|  |  |  |  |  | P = 0003 *** | Sh to AT+0.01 |  |
|  |  |  |  |  | P < 0001 **** | Sh to AT+0.05 |  |
|  |  |  |  |  | P = 0.0054 *** | Sh vs. AT+0.1 |  |
|  |  |  |  | P = 0.746 n.s. | n.s. | AT to AT+0.01 |  |
|  |  |  |  |  |  | AT to AT+0.05 |  |
|  |  |  |  |  |  | AT to AT+0.1 |  |
|  |  |  |  |  |  | AT+0.05 to AT+0.1 |  |
|  |  |  |  |  |  | AT+0.01 to AT+0.05 |  |
|  |  |  |  |  |  | AT+0.01 to AT+0.1 |  |
|  |  |  |  |  |  |  |  |
|  |  |  |  | **Two-way ANOVA** | **Dunn’s multiple comparison** | **Groups Compared** | **Frequency range** |
|  |  | **f-ABR** |  | P<0.0001 **** | P < 0.0001 **** | Sh to AT | 8-32 kHz |
|  |  |  |  | F(4.567) = 72.45 | P < 0.0001 **** | Sh to AT+0.01 | 8-32 kHz |
|  |  |  |  |  | P < 0.0001 **** | Sh to AT+0.05 | 8-32 kHz |
|  |  |  |  |  | P < 0.0001 **** | Sh vs. AT+0.1 | 8-32 kHz |
|  |  |  |  | P = 0.251 (n.s.) | n.s. | AT to AT+0.01 |  |
|  |  |  |  | F(3,414) = 1.371 |  | AT to AT+0.05 |  |
|  |  |  |  |  |  | AT to AT+0.1 |  |
|  |  |  |  |  |  | AT+0.05 to AT+0.1 |  |
|  |  |  |  |  |  | AT+0.01 to AT+0.05 |  |
|  |  |  |  |  |  | AT+0.01 to AT+0.1 |  |
|  |  |  |  |  |  |  |  |

|  |  |  |  | **Kruskal-Wallis** | **Dunn’s multiple comparison** | **Groups Compared** |  |
| --- | --- | --- | --- | --- | --- | --- | --- |
| **14d post AT + 6 weeks** | | **Click** |  | P<0.0001 **** | P = 0.0122 ** | Sh to AT |  |
| **(Figure 1B)** |  |  |  |  | P < 0001 **** | Sh to AT+0.05 |  |
|  |  |  |  |  | P = 0.002 ** | Sh vs. AT+0.1 |  |
|  |  |  |  | P =0.1205 (n.s.) | n.s. | AT to AT+0.05 |  |
|  |  |  |  |  |  | AT to AT+0.1 |  |
|  |  |  |  |  |  | AT+0.05 to AT+0.1 |  |
|  |  | **Noise** |  | P=0.0002 *** | P =0.008 **** | Sh to AT |  |
|  |  |  |  |  | P < 0001 **** | Sh to AT+0.05 |  |
|  |  |  |  |  | P = 0.0026 *** | Sh vs. AT+0.1 |  |
|  |  |  |  | P = 0.2216 n.s. | n.s. | AT to AT+0.05 |  |
|  |  |  |  |  |  | AT to AT+0.1 |  |
|  |  |  |  |  |  | AT+0.05 to AT+0.1 |  |
|  |  |  |  |  |  |  |  |
|  |  |  |  | **Two-way ANOVA** | **Dunn’s multiple comparison** | **Groups Compared** | **Frequency range** |
|  |  | **f-ABR** |  | P<0.0001 **** | P < 0.0001******** | Sh to AT | 8-32 kHz |
|  |  |  |  | F(3,288) = 103.7 | P < 0.0001******** | Sh to AT+0.05 | 8-32 kHz |
|  |  |  |  |  | P < 0.0001******** | Sh vs. AT+0.1 | 8-32 kHz |
|  |  |  |  |  |  | AT to AT+0.05 |  |
|  |  |  |  |  |  | AT to AT+0.1 |  |
|  |  |  |  | P = 0.110 (n.s.) | n.s. | AT+0.05 to AT+0.1 |  |
|  |  |  |  |  |  |  |  |
|  |  |  |  |  |  |  |  |
|  |  |  |  |  |  |  |  |
|  |  |  |  |  |  |  |  |
| **(Figure 2)** | no statistics |  |  |  |  |  |  |
|  |  |  |  |  |  |  |  |
|  |  |  |  |  |  |  |  |

|  | **Brain oscillation** | **Groups compared** | **Stimulus range** | **EVOKED LFP activity** | **(Auditory Cortex)** | **INDUCED LFP activity** | **Sidak’s multiple** |  |
| --- | --- | --- | --- | --- | --- | --- | --- | --- |
|  |  |  |  | **2-way RM-ANOVA** (factor exp. group) | **Sidak’s multiple comparison test** (adjusted P-value) | **2-way ANOVA** (factor exp. group) | **comparison** (adjusted P-value) |  |
| **14d post AT** |  |  | 4.7 - 6.7 kHz | P=0.0072 ** | P=0.0107 * | P=0.1077 (*) | P=0.1006 (*) |  |
| **(Figure 3A)** | alpha (5-15Hz) | **Sh to AT** | 10.3 - 12.3 kHz | F(1,14)=9.896 | P=0.0482 * | F(1,14)=2.953 | P=0.3236 n.s. |  |
|  |  |  | 21.7 - 23.7 kHz |  | P=0.0112 * |  | P=0.7574 n.s. |  |
|  |  |  | 4.7 - 6.7 kHz | P=0.0154 * | P=0.0449 * | P=0.1480 n.s. | P=0.1143 n.s. |  |
|  |  | **Sh to AT+0.01** | 10.3 - 12.3 kHz | F(1,13)=7.773 | P=0.0240 * | F(1,13)=2.366 | P=0.3325 n.s. |  |
|  |  |  | 21.7 - 23.7 kHz |  | P=0.0033 ** |  | P=0.8705 n.s. |  |
|  |  |  | 4.7 - 6.7 kHz | P=0.9564 n.s. | P=0.9989 n.s. | P=0.9367 n.s. | P=0.9974 n.s. |  |
|  |  | **Sh to AT+0.05** | 10.3 - 12.3 kHz | F(1,13)=0.003110 | P>0.9999 n.s. | F(1,13)=0.006554 | P>0.9999 n.s. |  |
|  |  |  | 21.7 - 23.7 kHz |  | P=0.9790 n.s. |  | P=0.9998 n.s. |  |
|  |  |  | 4.7 - 6.7 kHz | P=0.6229 n.s. | P=0.9982 n.s. | P=0.8045 n.s. | P=0.9998 n.s. |  |
|  |  | **Sh to AT+0.1** | 10.3 - 12.3 kHz | F(1,12)=0.2547 | P=0.4852 n.s. | F(1,12)=0.06407 | P=0.7773 n.s. |  |
|  |  |  | 21.7 - 23.7 kHz |  | P=0.5196 n.s. |  | P>0.9999 n.s. |  |
| **(Figure 3B)** | beta (15-25Hz) |  | 4.7 - 6.7 kHz | P=0.0056 ** | P=0.0028 ** | P=0.0600 (*) | P=0.0557 (*) |  |
|  |  | **Sh to AT** | 10.3 - 12.3 kHz | F(1,14)=10.70 | P=0.0642 (*) | F(1,14)=4.187 | P=0.1991 n.s. |  |
|  |  |  | 21.7 - 23.7 kHz |  | P=0.0391 * |  | P=0.6588 n.s. |  |
|  |  |  | 4.7 - 6.7 kHz | P=0.0063 ** | P=0.0034 ** | P=0.0823 (*) | P=0.0651 (*) |  |
|  |  | **Sh to AT+0.01** | 10.3 - 12.3 kHz | F(1,13)=10.60 | P=0.0197 * | F(1,13)=3.544 | P=0.1715 n.s. |  |
|  |  |  | 21.7 - 23.7 kHz |  | P=0.0504 (*) |  | P=0.8253 n.s. |  |
|  |  |  | 4.7 - 6.7 kHz | P=0.4773 n.s. | P=0.9998 n.s. | P=0.4087 n.s. | P=0.6437 n.s. |  |
|  |  | **Sh to AT+0.05** | 10.3 - 12.3 kHz | F(1,13)=0.5355 | P>0.9999 n.s. | F(1,13)=0.7289 | P=0.711 n.s. |  |
|  |  |  | 21.7 - 23.7 kHz |  | P=0.9790 n.s. |  | P=0.9999 n.s. |  |
|  |  |  | 4.7 - 6.7 kHz | P=0.2086 n.s. | P=0.9982 n.s. | P=0.3438 n.s. | P=0.881 n.s. |  |
|  |  | **Sh to AT+0.1** | 10.3 - 12.3 kHz | F(1,12)=1.766 | P>0.4852 n.s. | F(1,12)=0.9714 | P=0.2389 n.s. |  |
|  |  |  | 21.7 - 23.7 kHz |  | P=0.5196 n.s. |  | P=0.9789 n.s. |  |
| **(Figure 3C)** | low gamma (25-35Hz) | | 4.7 - 6.7 kHz | P=0.0204 * | P=0.0261 * | P=0.1113 n.s. | P=0.1749 n.s. |  |
|  |  | **Sh to AT** | 10.3 - 12.3 kHz | F(1,14)=6.835 | P=0.1531 n.s. | F(1,14)=2.890 | P=0.2836 n.s. |  |
|  |  |  | 21.7 - 23.7 kHz |  | P=0.0262 * |  | P=0.7614 n.s. |  |
|  |  |  | 4.7 - 6.7 kHz | P=0.0089 ** | P=0.0073 ** | P=0.2749 n.s. | P=0.2251 n.s. |  |
|  |  | **Sh to AT+0.01** | 10.3 - 12.3 kHz | F(1,13)=9.442 | P=0.0337 * | F(1,13)=1.299 | P=0.6058 n.s. |  |
|  |  |  | 21.7 - 23.7 kHz |  | P=0.0331 * |  | P=0.9998 n.s. |  |
|  |  |  | 4.7 - 6.7 kHz | P=0.7341 n.s. | P=0.8718 n.s. | P=0.4963 n.s. | P=0.788 n.s. |  |
|  |  | **Sh to AT+0.05** | 10.3 - 12.3 kHz | F(1,13)=0.1205 | P=0.9810 n.s. | F(1,13)=0.4900 | P=0.8125 n.s. |  |
|  |  |  | 21.7 - 23.7 kHz |  | P=0.9707 n.s. |  | P=0.9995 n.s. |  |
|  |  |  | 4.7 - 6.7 kHz | P=0.2812 n.s. | P=0.9674 n.s. | P=0.4317 n.s. | P=0.8769 n.s. |  |
|  |  | **Sh to AT+0.1** | 10.3 - 12.3 kHz | F(1,12)=1.273 | P=0.1662 n.s. | F(1,12)=0.6620 | P=0.4727 n.s. |  |
|  |  |  | 21.7 - 23.7 kHz |  | P=0.3419 n.s. |  | P=0.9947 n.s. |  |
| **(Figure 3D)** | mid gamma (35-65Hz) | | 4.7 - 6.7 kHz | P=0.3773 n.s. | P=0.8630 n.s. | P=0.4869 n.s. | P=0.8672 n.s. |  |
|  |  | **Sh to AT** | 10.3 - 12.3 kHz | F(1,14)=0.8314 | P=0.7833 n.s. | F(1,14)=0.5100 | P=0.8371 n.s. |  |
|  |  |  | 21.7 - 23.7 kHz |  | P=0.5978 n.s. |  | P=0.9384 n.s. |  |
|  |  |  | 4.7 - 6.7 kHz | P=0.0213 * | P=0.1050 (*) | P=0.2393 n.s. | P=0.3337 n.s. |  |
|  |  | **Sh to AT+0.01** | 10.3 - 12.3 kHz | F(1,13)=6.847 | P=0.0359 * | F(1,13)=1.521 | P=0.3956 n.s. |  |
|  |  |  | 21.7 - 23.7 kHz |  | P=0.0158 * |  | P=0.9646 n.s. |  |
|  |  |  | 4.7 - 6.7 kHz | P=0.7528 n.s. | P=0.9904 n.s. | P=0.5016 n.s. | P=0.9384 n.s. |  |
|  |  | **Sh to AT+0.05** | 10.3 - 12.3 kHz | F(1,13)=0.1035 | P=0.9996 n.s. | F(1,13)=0.4778 | P=0.9760 n.s. |  |
|  |  |  | 21.7 - 23.7 kHz |  | P=0.9570 n.s. |  | P=0.7059 n.s. |  |
|  |  |  | 4.7 - 6.7 kHz | P=0.8157 n.s. | P=0.9666 n.s. | P=0.7147 n.s. | P=0.9924 n.s. |  |
|  |  | **Sh to AT+0.1** | 10.3 - 12.3 kHz | F(1,12)=0.05676 | P=0.3556 n.s. | F(1,12)=0.1401 | P>0.9999 n.s. |  |
|  |  |  | 21.7 - 23.7 kHz |  | P=0.8864 n.s. |  | P=0.932 n.s. |  |
| **(Figure 3E)** | high gamma (65-125Hz) |  | 4.7 - 6.7 kHz | P=0.7242 n.s. | P=0.9867 n.s. | P=0.8641 n.s. | P>0.9998 n.s. |  |
|  |  | **Sh to AT** | 10.3 - 12.3 kHz | F(1,14)=0.1296 | P=0.9726 n.s. | F(1,14)=0.03040 | P=0.9907 n.s. |  |
|  |  |  | 21.7 - 23.7 kHz |  | P=0.3202 n.s. |  | P=0.9854 n.s. |  |
|  |  |  | 4.7 - 6.7 kHz | P=0.0434 * | P=0.2481 n.s. | P=0.9472 n.s. | P=0.9987 n.s. |  |
|  |  | **Sh to AT+0.01** | 10.3 - 12.3 kHz | F(1,13)=5.004 | P=0.1069 n.s. | F(1,13)=0.004557 | P=0.9996 n.s. |  |
|  |  |  | 21.7 - 23.7 kHz |  | P=0.0647 n.s. |  | P=0.9963 n.s. |  |
|  |  |  | 4.7 - 6.7 kHz | P=0.7366 n.s. | P=0.9926 n.s. | P=0.8032 n.s. | P=0.9189 n.s. |  |
|  |  | **Sh to AT+0.05** | 10.3 - 12.3 kHz | F(1,13)=0.1181 | P=0.9495 n.s. | F(1,13)=0.06468 | P=0.9940 n.s. |  |
|  |  |  | 21.7 - 23.7 kHz |  | P=0.9982 n.s. |  | P>0.9999 n.s. |  |
|  |  |  | 4.7 - 6.7 kHz | P=0.4459 n.s. | P=0.6645 n.s. | P=0.9670 n.s. | P>0.9999 n.s. |  |
|  |  | **Sh to AT+0.1** | 10.3 - 12.3 kHz | F(1,12)=0.6212 | P=0.9697 n.s. | F(1,12)=0.001787 | P=0.9795 n.s. |  |
|  |  |  | 21.7 - 23.7 kHz |  | P=0.9996 n.s. |  | P=0.9930 n.s. |  |
|  | **Brain oscillation** | **Groups compared** | **Stimulus range** | **EVOKED LFP activity** | **(Auditory Cortex)** | **INDUCED LFP activity** | **Sidak’s multiple** |  |
|  |  |  |  | **2-way RM-ANOVA** (factor exp. group) | **Sidak’s multiple comparison test** (adjusted P-value) | **2-way ANOVA** (factor exp. group) | **comparison** (adjusted P-value) |  |
|  |  |  |  |  |  |  |  |  |
|  | **Brain oscillation** | **Groups compared** |  | **2-way RM-ANOVA (factor exp. group)** | **Sidak’s multiple comparison test (adjusted P-value)** | **EVOKED ASSR 8 kHz responses (Auditory Cortex)** |  |  |
| **14d post AT** |  | **Sh to AT** |  | P=0.0090 ** | 40 Hz P=0.0095 |  |  |  |
| **(Figure 4A)** | alpha |  |  | F(1,14)=9.192 |  |  |  |  |
|  |  | **Sh to AT+0.01** |  | P=0.0164 * | n.s. |  |  |  |
|  |  |  |  | F(1,13)=7.585 |  |  |  |  |
|  |  | **Sh to AT+0.05** |  | P=0.8821 n.s. |  |  |  |  |
|  |  |  |  | F(1,13)=0.02289 |  |  |  |  |
|  |  | **Sh to AT+0.1** |  | P=0.3202 n.s. |  |  |  |  |
|  |  |  |  | F(1,12)=1.075 |  |  |  |  |
| **(Figure 4B)** | beta | **Sh to AT** |  | P=0.0128 ** | n.s. |  |  |  |
|  |  |  |  | F(1,14)=8.125 |  |  |  |  |
|  |  | **Sh to AT+0.01** |  | P=0.0207 * | n.s. |  |  |  |
|  |  |  |  | F(1,13)=6.926 |  |  |  |  |
|  |  | **Sh to AT+0.05** |  | P=0.7448 n.s. |  |  |  |  |
|  |  |  |  | F(1,13)=0.1106 |  |  |  |  |
|  |  | **Sh to AT+0.1** |  | P=0.0955 n.s. | n.s. |  |  |  |
|  |  |  |  | F(1,12)=3.273 |  |  |  |  |
| **(Figure 4C)** | low gamma | **Sh to AT** |  | P=0.0273 * | n.s. |  |  |  |
|  |  |  |  | F(1,14)=6.072 |  |  |  |  |
|  |  | **Sh to AT+0.01** |  | P=0.0362* | n.s. |  |  |  |
|  |  |  |  | F(1,13)=5.452 |  |  |  |  |
|  |  | **Sh to AT+0.05** |  | P=0.4794 n.s. |  |  |  |  |
|  |  |  |  | F(1,13)=0.5304 |  |  |  |  |
|  |  | **Sh to AT+0.1** |  | P=0.1477 n.s. |  |  |  |  |
|  |  |  |  | F(1,12)=2.395 |  |  |  |  |
| **(Figure 4D)** | mid gamma | **Sh to AT** |  | P=0.0697 (*) | 40Hz P=0.0014 |  |  |  |
|  |  |  |  | F(1,14)=3.857 |  |  |  |  |
|  |  | **Sh to AT+0.01** |  | P=0.0489 * | 40Hz P=0.0009 |  |  |  |
|  |  |  |  | F(1,13)=3.857 |  |  |  |  |
|  |  | **Sh to AT+0.05** |  | P=0.3871 n.s. |  |  |  |  |
|  |  |  |  | F(1,13)=0.80095 |  |  |  |  |
|  |  | **Sh to AT+0.1** |  | P=0.1428 n.s. |  |  |  |  |
|  |  |  |  | F(1,12)=2.459 |  |  |  |  |
| **(Figure 4E)** | high gamma | **Sh to AT** |  | P=0.1517 n.s. |  |  |  |  |
|  |  |  |  | F(1,14)=2.299 |  |  |  |  |
|  |  | **Sh to AT+0.01** |  | P=0.1575 n.s. |  |  |  |  |
|  |  |  |  | F(1,13)=2.250 |  |  |  |  |
|  |  | **Sh to AT+0.05** |  | P=0.6426 n.s. |  |  |  |  |
|  |  |  |  | F(1,13)=0.2257 |  |  |  |  |
|  |  | **Sh to AT+0.1** |  | P=0.2516 n.s. |  |  |  |  |
|  |  |  |  | F(1,12)=1.451 |  |  |  |  |
|  |  |  |  |  |  |  |  |  |
| **14d post AT** | **Trauma effect** | **Sh to AT** |  | P<0.0001 **** | ≤ 51dB n.s |  |  |  |
| **(Figure 5A)** |  |  |  | F(1,787)=571.8 | 54dB P=0.0111 * |  |  |  |
|  |  |  |  |  | 57dB P=0.0017 * |  |  |  |
|  |  |  |  |  | ≥60dB P<0.0001 * |  |  |  |
|  |  | **AT to AT+0.05** |  | P<0.0001 **** | ≤ 90dB n.s |  |  |  |
|  |  |  |  | F(1,596)=25.47 | 93dB P=0.0168 * |  |  |  |
|  |  |  |  |  | 96dB P=0.0055* |  |  |  |
|  |  |  |  |  | 99 dBP=0.0049 ** |  |  |  |
|  |  | **AT to AT+0.1** |  | P=0.0014 ** | n.s. |  |  |  |
|  |  |  |  | F(1,465)=10.29 |  |  |  |  |
| **(Figure 5C)** | **Loss of** | **Sh to AT** |  | P<0.0001 **** | ≤ 54dB n.s. |  |  |  |
|  | **masking effect** |  |  | F(1,787)=549.1 | 57dB P=0.0407 ** |  |  |  |
|  | (Pre - 14d) |  |  |  | 60dB P=0.0028 |  |  |  |
|  |  |  |  |  | ≥63dB P<0.0001 |  |  |  |
|  |  | **Sh to AT+0.05** |  | P<0.0001 **** | ≤ 54dB n.s |  |  |  |
|  |  |  |  | F(1,793)=364.1 | 57dB P=0.0469. |  |  |  |
|  |  |  |  |  | 60dB P=0.0063. |  |  |  |
|  |  |  |  |  | ≥63dB P<0.0001. |  |  |  |
|  |  | **Sh to AT+0.1** |  | P<0.0001 **** | ≤ 57dB n.s |  |  |  |
|  |  |  |  | F(1,662)=186.8 | 60dB P=0.0289. |  |  |  |
|  |  |  |  |  | 63dB P=0.0012 |  |  |  |
|  |  |  |  |  | ≥66dB P=0.0001. |  |  |  |
|  |  |  |  |  |  |  |  |  |
|  |  | **Groups compared** |  | **2-way RM-ANOVA** (factor exp. group) | **Sidak’s multiple comparison test** (adjusted P-value) | **EVOKED ASSR 8 kHz responses (Auditory Cortex)** |  |  |
| **14d post AT** | **Loss of** | **AT to AT+0.05** |  | P<0.0001 **** | ≤ 90dB n.s |  |  |  |
| **(Figure 5C)** | **masking effect** |  |  | F(1,596)=18.37 | 93dB P=0.0153 |  |  |  |
|  | (Pre - 14d) |  |  |  | 96dB P=0.001 |  |  |  |
|  |  |  |  |  | 99dB P=0.0027. |  |  |  |
|  |  | **AT to AT+0.1** |  | P<0.0001 **** | ≤ 90dB n.s |  |  |  |
|  |  |  |  | F(1,465)=26.55 | 93dB P=0.0002 |  |  |  |
|  |  |  |  |  | 96dB P<0.0001 |  |  |  |
|  |  |  |  |  | 99dB P=0.0001. |  |  |  |
|  |  |  |  |  |  |  |  |  |
|  |  |  |  |  |  |  |  |  |
|  | **Inner hair cell ribbon number** | **Cochlear turn** |  | **Kruskal-Wallis** | **Dunn’s multiple comparison** | **Groups Compared** |  |  |
| **14d post AT** |  | **apical** |  | P = 0.2797 (n.s.) | n.s. | Sh to AT |  |  |
| **(Figure 6B)** |  |  |  |  |  | Sh to AT+0.01 |  |  |
|  |  |  |  |  |  | Sh to AT+0.05 |  |  |
|  |  |  |  |  |  | Sh vs. AT+0.1 |  |  |
|  |  |  |  | P = 0.7949 (n.s.) | n.s. | AT to AT 0.01 |  |  |
|  |  |  |  |  |  | AT to AT 0.05 |  |  |
|  |  |  |  |  |  | AT to AT 0.1 |  |  |
|  |  |  |  |  |  | AT 0.01 to AT 0.05 |  |  |
|  |  |  |  |  |  | AT 0.01 to AT 0.1 |  |  |
|  |  |  |  |  |  | AT 0.05 to AT 0.1 |  |  |
|  |  | **medial** |  | P = 0.002 ** | 0.0005 *** | Sh to AT |  |  |
|  |  |  |  |  | 0.0376 * | Sh to AT+0.01 |  |  |
|  |  |  |  |  | 0.5134 n.s. | Sh to AT+0.05 |  |  |
|  |  |  |  |  | >0.9999 n.s. | Sh vs. AT+0.1 |  |  |
|  |  |  |  | P = 0.0893 (n.s.) | n.s. | AT to AT 0.01 |  |  |
|  |  |  |  |  |  | AT to AT 0.05 |  |  |
|  |  |  |  |  |  | AT to AT 0.1 |  |  |
|  |  |  |  |  |  | AT 0.01 to AT 0.05 |  |  |
|  |  |  |  |  |  | AT 0.01 to AT 0.1 |  |  |
|  |  |  |  |  |  | AT 0.05 to AT 0.1 |  |  |
|  |  | **midbasal** |  | P < 0.0001 **** | <0.0001 **** | Sh to AT |  |  |
|  |  |  |  |  | <0.0001**** | Sh to AT+0.01 |  |  |
|  |  |  |  |  | 0.2091 n.s. | Sh to AT+0.05 |  |  |
|  |  |  |  |  | 0.0004 *** | Sh vs. AT+0.1 |  |  |
|  |  |  |  | P = 0.0073 ** | >0.9999 n.s. | AT to AT 0.01 |  |  |
|  |  |  |  |  | 0.0171 * | AT to AT 0.05 |  |  |
|  |  |  |  |  | >0.9999 n.s. | AT to AT 0.1 |  |  |
|  |  |  |  |  | 0.0182 * | AT 0.01 to AT 0.05 |  |  |
|  |  |  |  |  | >0.9999 n.s. | AT 0.01 to AT 0.1 |  |  |
|  |  |  |  |  | 0.1013 n.s. | AT 0.05 to AT 0.1 |  |  |
|  |  | **basal** |  | P < 0.0001 **** | <0.0001 **** | Sh to AT |  |  |
|  |  |  |  |  | <0.0001 **** | Sh to AT+0.01 |  |  |
|  |  |  |  |  | 0.2917 n.s. | Sh to AT+0.05 |  |  |
|  |  |  |  |  | 0.1269 n.s. | Sh vs. AT+0.1 |  |  |
|  |  |  |  | P = 0.0014 ** | >0.9999 n.s. | AT to AT 0.01 |  |  |
|  |  |  |  |  | 0.0097 ** | AT to AT 0.05 |  |  |
|  |  |  |  |  | 0.1151 n.s. | AT to AT 0.1 |  |  |
|  |  |  |  |  | 0.014 * | AT 0.01 to AT 0.05 |  |  |
|  |  |  |  |  | 0.113 n.s. | AT 0.01 to AT 0.1 |  |  |
|  |  |  |  |  | >0.9999 n.s. | AT 0.05 to AT 0.1 |  |  |
|  |  |  |  |  |  |  |  |  |
|  |  |  |  |  |  |  |  |  |
|  |  |  |  |  |  |  |  |  |
|  |  |  |  | **One-way ANOVA** | **Dunnett’s / Tukey´s multiple comp.** | **Groups Compared** |  |  |
| **14d post AT + 6 weeks** | **apical** |  | P = 0.3892 (n.s.) | n.s. | Sh to AT |  |  |  |
| **(Figure 6D)** |  |  |  |  |  | Sh to AT+0.01 |  |  |
|  |  |  |  |  |  | Sh to AT+0.05 |  |  |
|  |  |  |  |  |  | Sh vs. AT+0.1 |  |  |
|  |  |  |  | P = 0.2409 (n.s.) | n.s. | AT to AT 0.05 |  |  |
|  |  |  |  |  |  | AT to AT 0.1 |  |  |
|  |  |  |  |  |  | AT 0.05 to AT 0.1 |  |  |
|  |  | **medial** |  | P = 0.0071 ** | 0.1014 n.s. | Sh to AT |  |  |
|  |  |  |  |  | 0.7107 n.s. | Sh to AT+0.05 |  |  |
|  |  |  |  |  | 0.0036 ** | Sh vs. AT+0.1 |  |  |
|  | |  |  |  | P = 0.0260 * | 0.3747 n.s. | AT to AT 0.05 |  |
|  |  |  |  |  | 0.2357 n.s. | AT to AT 0.1 |  |  |
|  |  |  |  |  | 0.0201 * | AT 0.05 to AT 0.1 |  |  |
|  |  | **midbasal** |  | P = 0.0065 ** | 0.0020 ** | Sh to AT |  |  |
|  |  |  |  |  | 0.0549 n.s. | Sh to AT+0.05 |  |  |
|  |  |  |  |  | 0.0396 * | Sh vs. AT+0.1 |  |  |
|  |  |  |  | P = 0.1093 n.s. | 0.3829 n.s. | AT to AT 0.05 |  |  |
|  |  |  |  |  | 0.5389 n.s. | AT to AT 0.1 |  |  |
|  |  |  |  |  | 0.9720 n.s. | AT 0.05 to AT 0.1 |  |  |
|  |  | **basal** |  | P = 0.0004 *** | <0.0001 **** | Sh to AT |  |  |
|  |  |  |  |  | 0.0263 * | Sh to AT+0.05 |  |  |
|  |  |  |  |  | 0.0527 n.s. | Sh vs. AT+0.1 |  |  |
|  |  |  |  | P = 0.0358 * | 0.0650 n.s. | AT to AT 0.05 |  |  |
|  |  |  |  |  | 0.0518 n.s. | AT to AT 0.1 |  |  |
|  |  |  |  |  | 0.9747 n.s. | AT 0.05 to AT 0.1 |  |  |
|  |  |  |  |  |  |  |  |  |
|  |  |  |  |  |  |  |  |  |
|  |  | **ABR wave latency** |  | **Ordinary one-way ANOVA** | **Tukey's multiple comparisons** | **Groups Compared** |  |  |
| **14d post AT + 6 weeks** |  |  | P=0.004*** | P = 0.049 * | AT to AT+0.05 |  |  |  |
| **(Figure 6F)** |  | **Mean 20 – 45 dB SPL** |  | F(2,25) = 6.795 | P = 0.0041** | AT to AT+0.1 |  |  |
|  |  |  |  |  | n.s. | AT+0.05 to AT+0.1 |  |  |
|  |  |  |  |  |  |  |  |  |
|  |  |  |  |  |  |  |  |  |
|  |  |  |  |  |  |  |  |  |
|  |  |  |  |  |  |  |  |  |
|  |  |  |  |  |  |  |  |  |
|  |  |  |  |  |  |  |  |  |
|  |  | **Mulit-Click Pulse Resp**. |  | **Ordinary one-way ANOVA** | **Dunnett's multiple comparisons** | **Groups Compared** |  |  |
| **14d post AT** | |  |  |  | P = 0.045 * | P = 0.03 * | AT to AT+0.05 |  |
| **(Figure 7A)** |  | **y (x=2.5ms)** |  | F(2,37) = 3.370 | n.s. | AT to AT+0.01 |  |  |
|  |  |  |  |  |  |  |  |  |
|  |  |  |  |  |  |  |  |  |
| **14d post AT + 6 weeks** |  |  | P = 0.0488 * | P = 0.0358 * | AT to AT+0.05 |  |  |  |
| **(Figure 7B)** |  | **y (x=2.5ms)** |  | F(2,25) = 3.415 | n.s. | AT to AT+0.01 |  |  |
|  |  |  |  |  |  |  |  |  |
|  | **Evoked ASSR amp.** | **groups compared** |  | **2-way RM-ANOVA** (factor exp. group) | **Sidak’s multiple comparison test** (adjusted P-value) | **Modulation frequency** |  |  |
| **14d post AT** |  | **Sh to AT+V** |  | P = 0.0098 ** | P = 0.0322* | mf 20 Hz |  |  |
| **(Suppl. Figure 5A)** |  |  |  | F (1, 14) = 8.928 |  |  |  |  |
|  |  | **Sh to AT+0.01** |  | P = 0.0020 ** | P = 0.0137* | mf 32 Hz |  |  |
|  |  |  |  | F (1, 13) = 14.87 | P = 0.024* | mf 40 Hz |  |  |
|  |  |  |  |  | P = 0.0103* | mf 80 Hz |  |  |
|  |  |  |  |  | P = 0.0103* | mf 160 Hz |  |  |
|  |  |  |  |  | P = 0.0241* | mf 320 Hz |  |  |
|  |  | **Sh to AT+0.05** |  | P = 0.0192 * | all freq. n.s. |  |  |  |
|  |  |  |  | F (1, 13) = 7.141 |  |  |  |  |
|  |  | **Sh to AT+0.1** |  | P = 0.2549 n.s. | all freq. n.s. |  |  |  |
|  |  |  |  | F (1, 12) = 1.430 |  |  |  |  |
|  |  | **AT to AT+0.01** |  | P = 0.6343 n.s. | all freq. n.s. |  |  |  |
|  |  |  |  | F (1, 11) = 0.2393 |  |  |  |  |
|  |  | **AT to AT+0.05** |  | P = 0.9854 n.s. | all freq. n.s. |  |  |  |
|  |  |  |  | F (1, 11) = 0.0003524 |  |  |  |  |
|  |  | **AT to AT+0.1** |  | P = 0.1311 n.s. | all freq. n.s. |  |  |  |
|  |  |  |  | F (1, 10) = 2.705 |  |  |  |  |
|  |  | **AT+0.01 to AT+0.05** |  | P = 0.6580 n.s. | all freq. n.s. |  |  |  |
|  |  |  |  | F (1, 10) = 0.2081 |  |  |  |  |
|  |  | **AT+0.01 to AT+0.1** |  | P = 0.0274 * | all freq. n.s. |  |  |  |
|  |  |  |  | F (1, 9) = 6.915 |  |  |  |  |
|  |  | **AT+0.05 to AT+0.1** |  | P = 0.1835 n.s. | all freq. n.s. |  |  |  |
|  |  |  |  | F (1, 9) = 2.076 |  |  |  |  |
|  |  |  |  |  |  |  |  |  |

Sh: Sham + Veh

AT: AT + Veh

AT+0.01: AT + 0.01mg/kg DX243

AT+0.05: AT + 0.05mg/kg DX243

AT+0.1: AT + 0.1mg/kg DX243

Statistically significant difference: ***** p < 0.05, ****** p < 0.01, ******* p < 0.001

n.s. not significant (p > 0.05)

(*) trend (0.05 < p < 0.11)

RM-ANOVA (repeated measurement ANOVA)
