# Supplementary material for: DX243 counteracts both acoustic trauma-induced reduction in cortical brain oscillations and cochlear synaptopathy
Source: Front Pharmacol. 2026 Jan 14;16:1673189. doi: 10.3389/fphar.2025.1673189 (PMC12847371; doi:10.3389/fphar.2025.1673189)
Supplement: Supplementary file 8 [file Table3.docx]

**Supplementary Table 3:** Fit parameter for Figure 7. Data are indicated as mean±SD values of the parameters determined for individual ears in the investigated groups. The value of a given parameter of a given ear is calculated as the mean value of that given parameter gained be the individual fit process of data measured by click-stimulus trains of three, four and five click stimuli.

| **14d post AT** (Mean±SD) | | | | | |  |  |
| --- | --- | --- | --- | --- | --- | --- | --- |
|  | **Sham+Veh** | **AT+Veh** | **AT+0.01mg** | **AT+0.05mg** | **AT+0.1mg** | | |
| **n** | 18 | 16 | 12 | 12 | 10 | | |
| **p** | 0.14±0.05 | 0.17±0.07 | 0.16±0.08 | 0.09±0.05 | 0.11±0.09 | | |
| **m1 (ms)** | 9.8±1.8 | 8.3±2.0 | 8.1±1.7 | 7.7±2.9 | 8.4±2.6 | | |
| **m2 (ms)** | 0.90±0.13 | 1.14±0.16 | 1.14±0.17 | 1.15±0.15 | 1.09±0.13 | | |
| **s1 (ms)** | 1.54±0.83 | 1.34±0.79 | 1.27±0.91 | 1.34±0.49 | 1.47±0.75 | | |
| **s2 (ms)** | 0.78±0.19 | 0.70±0.21 | 0.65±0.26 | 0.62±0.21 | 0.57±0.22 | | |
| **14d + 6 weeks post AT** (Mean±SD) | | | | | | |  |
|  | **Sham+Veh** | **AT+Veh** | **AT+0.01mg** | **AT+0.05mg** | **AT+0.1mg** | | |
| **n** | 8 | 10 |  | 10 | 8 | | |
| **p** | 0.20±0.08 | 0.16±0.08 |  | 0.10±0.07 | 0.11±0.04 | | |
| **m1 (ms)** | 9.6±1.8 | 8.5±1.5 |  | 8.0±2.0 | 8.7±1.5 | | |
| **m2 (ms)** | 0.89±0.23 | 1.19±0.16 |  | 1.10±0.10 | 1.12±0.27 | | |
| **s1 (ms)** | 1.85±0.90 | 1.43±0.72 |  | 1.33±0.86 | 0.98±0.51 | | |
| **s2 (ms)** | 0.95±0.12 | 0.61±0.15 |  | 0.55±0.14 | 0.72±0.23 | | |
